# Protein adduction by multiple aldehydes contributes to α-synuclein aggregation and dopaminergic neuron loss in rat models of Parkinson's disease

**DOI:** 10.1016/j.redox.2026.104468

**Published:** 2026-11-02

**Authors:** Lin Chen, Haiping Deng, Ni Li, Ya He, Guohong Zhai, Hong Pan, Chao Fang, Fuguo Shi

**Affiliations:** a Key Laboratory of Basic Pharmacology of Ministry of Education & Joint International Research Laboratory of Ethnomedicine of Ministry of Education, Zunyi Medical University, Zunyi, 563003, China; b Department of Pharmacy, Tongren Hospital, Shanghai Jiao Tong University School of Medicine, Shanghai, 200025, China

**Keywords:** Protein adduction, Aldehydes, Dopaminergic neuron, α-Synuclein, Parkinson's disease

## Abstract

Dopaminergic neurodegeneration in the substantia nigra causes the motor symptoms of Parkinson's disease (PD). The mechanisms underlying this neurodegeneration remain unclear. Aldehydes exhibit high reactivity that can covalently modify functional proteins and cause cell damage. This study aimed to characterize protein adduction by endogenous aldehydes and investigate its role in dopaminergic neuron loss in PD. Forty free aldehydes were identified in rat models of PD induced by 6-hydroxydopamine (6-OHDA) and rotenone using chemical isotope labeling coupled with high-resolution mass spectrometry. Twenty-two aldehydes were found in the rat brain for the first time. In addition to lipid peroxidation, amine oxidation was the major source of aldehydes. Levels of 27 aldehyde-protein adducts in the substantia nigra and striatum were increased by 1- to 5-fold at 4–6 h after 6-OHDA injection. Consistently, most of these protein adducts were significantly increased in the rotenone model. Application of the trapping reagent phenelzine and the aldehyde dehydrogenase-2 activator Alda-1 reduced protein adduction by aldehydes and attenuated neuron loss induced by 6-OHDA injection. Twelve aldehydes covalently modified α-synuclein in 6-OHDA-treated rats, with the lysine residue in the GLSK peptide identified as the primary binding target. Aldehydes caused peptide crosslinking and aggregation of α-synuclein. Additive effects of covalent modification by aldehydes contributed to neuron death. Collectively, protein adduction by multiple aldehydes contributes to α-synuclein aggregation and dopaminergic neuron loss in rat models of PD. Multiple biogenic aldehydes, especially those from abnormal oxidation of amines, represent a novel class of toxic molecules that play a pathogenic role in PD.

## Highlights

• Forty biogenic aldehydes were found in the brains of rat PD models.
• Amine oxidation was a major source of aldehydes in the rat brain.
• Twelve aldehydes modified α-synuclein and caused protein crosslinking and aggregation.
• Protein adduction by multiple aldehydes was associated with dopaminergic neuron loss.
• Aldehydes showed “additive effects” in protein modification and neuron death.

## Graphical abstract

**Figure:**
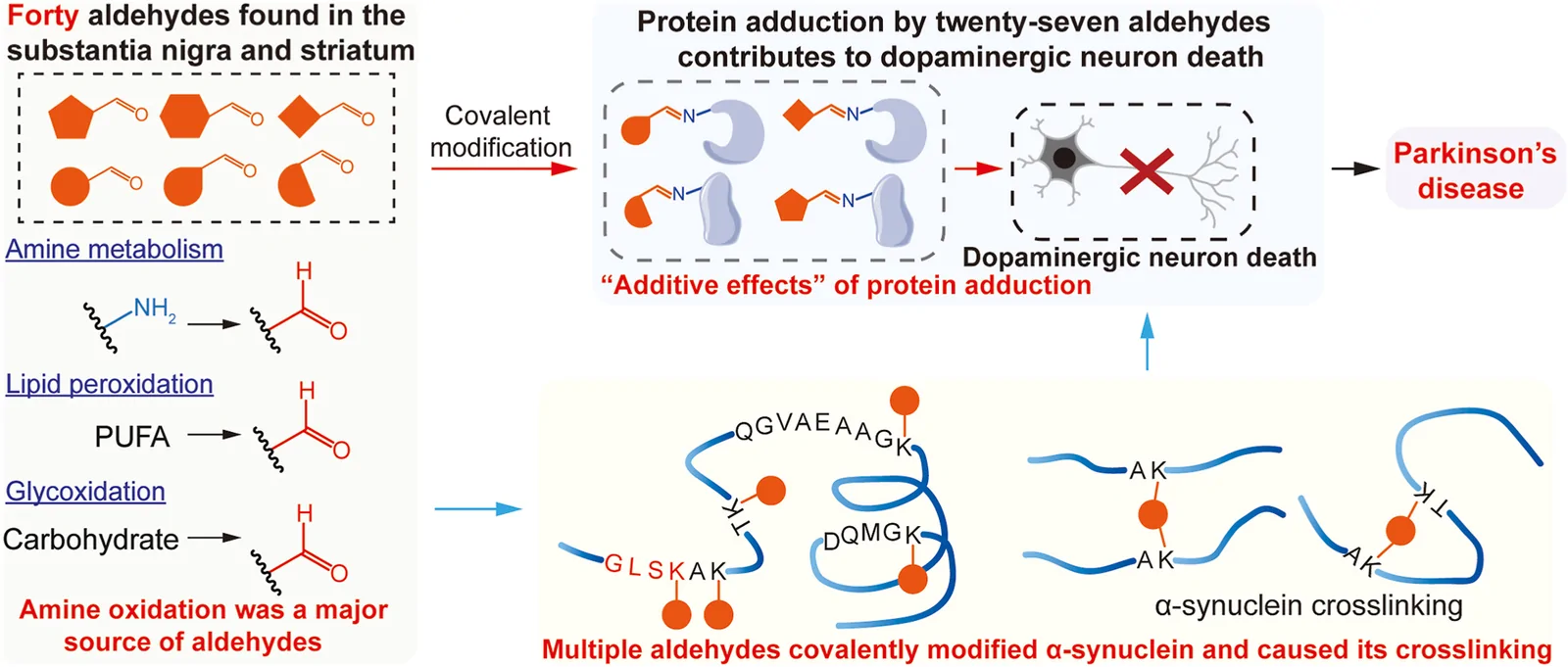

## 1 Introduction

Parkinson's disease (PD) is the second most prevalent neurodegenerative disease in the world. At present, no therapeutic intervention can slow down or arrest the disease progression of PD. The principal pathological hallmarks of PD are the selective loss of dopaminergic neurons in the substantia nigra (SN) pars compacta and the formation of Lewy bodies, which are primarily composed of aggregated α-synuclein, within surviving neurons [1]. The etiology of PD is considered multifactorial, with the majority of cases classified as sporadic with an undefined etiology [2]. The loss of dopaminergic neurons has been attributed to mitochondrial dysfunction, oxidative stress, neuroinflammation, and insufficient protein degradation [3]. However, the precise molecular events linking these diverse etiologies to dopaminergic neuron degeneration and α-synuclein aggregation remain incompletely understood.

Aldehydes are a large class of reactive and electrophilic carbonyl compounds that enable covalent modification of proteins and other biomolecules, resulting in cell injury or death [4]. Endogenous aldehydes are unstable intermediates generated during the metabolism of lipids, carbohydrates, amino acids, amines, and other molecules [5], [6]. Elevated levels of various aldehydes derived from lipid peroxidation, glycoxidation, and dopamine oxidation have been detected in the brains of PD patients and animal models. These include 4-hydroxynonenal (4-HNE), malondialdehyde (MDA), pyruvaldehyde, glyoxal, and 3,4-dihydroxyphenylacetaldehyde (DOPAL) [7], [8], [9], [10], [11], [12], [13], [14]. Notably, these aldehydes have been reported to covalently modify α-synuclein, an event that may promote α-synuclein aggregation by blocking lysine residues critical for ubiquitination [10]. DOPAL, an aberrant intermediate of dopamine metabolism, has garnered increasing research interest. Given that dopamine is the predominant neurotransmitter in nigrostriatal pathways, DOPAL accumulation has been proposed as a key mechanism underlying the selective degeneration of dopaminergic neurons [15]. Other aldehydes, such as acrolein, also possess neurotoxicity [8]. Inhibition of aldehyde dehydrogenases (ALDHs), a family of enzymes essential for aldehyde detoxification, causes aldehyde accumulation and represents a recognized pathogenic mechanism in PD [16]. Furthermore, inhibiting monoamine oxidase that oxidizes amines into aldehydes or directly scavenging reactive aldehydes have emerged as promising therapeutic strategies for PD [17]. These findings indicate that aldehydes may play a crucial role in PD pathogenesis.

Owing to their high reactivity, aldehydes readily form stable conjugates with nucleophiles. Consequently, the identification and quantification of endogenous aldehydes present considerable analytical challenges, and a substantial number of aldehyde species may remain unidentified due to their inherent instability. Chemical derivatization strategies, particularly those employing hydrazine-based reagents, are routinely used to stabilize the reactive carbonyl group for analysis [18]. Using isotopic/chemical labeling coupled with liquid chromatography-mass spectrometry (LC-MS), more than 138 fatty aldehydes derived from lipid peroxidation have been identified in rat brain tissue [19]. Beyond lipid peroxidation, a vast array of aldehydes also arises as metabolic intermediates from amine and hydroxyl oxidation, glycoxidation, carboxylic acid reduction, ester or amide hydrolysis, and other cellular biochemical processes [6], [20]. For instance, key neurotransmitters in the central nervous system, including dopamine, γ-aminobutyric acid (GABA), and 5-hydroxytryptamine, undergo oxidative deamination to yield their respective aldehyde intermediates: DOPAL, succinic semialdehyde, and 5-hydroxyindoleacetaldehyde. We therefore hypothesized that a much broader spectrum of reactive aldehyde intermediates, originating not solely from lipid peroxidation but also from amine oxidation, may undergo aberrant accumulation during PD pathogenesis. These aldehydes may subsequently engage in covalent protein modification, thereby contributing to dopaminergic neuron loss and α-synuclein aggregation.

This study aimed to (1) characterize protein adduction by endogenous aldehydes in the SN and striatum of both the 6-hydroxydopamine (6-OHDA)- and rotenone-induced rat models of PD; (2) delineate the temporal profiles of aldehyde–protein adduction ; (3) define the relationship between aldehyde–protein adduction and dopaminergic neuron loss ; (4) examine the covalent modification of α-synuclein by aldehydes and the formation of putative peptide crosslinks; and (5) explore the potential additive effects of covalent modifications by individual aldehydes and their collective contribution to dopaminergic neuron death.

## 2 Materials and methods

### 2.1 Chemicals and reagents

All chemicals and reagents were obtained from commercial sources and the details are shown in supplemental materials.

### 2.2 Identification of free aldehydes by chemical isotope labeling and their conjugates with free amino acids

Rat brain samples were collected after euthanasia, and then the striatum and SN regions were immediately and rapidly dissected on ice followed by freezing in liquid nitrogen. Specimens were homogenized in an ice bath with ice-cold 0.9% NaCl solution at a ratio of 1:3 (g:mL). The entire homogenization process was carried out gently and intermittently to minimize heat generation, and was completed in less than 5 min. The homogenates were immediately centrifuged at 14000 × g for 10 min at 4 °C. Then, the supernatant was immediately mixed with three volumes of ice-cold methanol to precipitate proteins, and the mixture was centrifuged again under the same conditions. The resulting supernatants were used for the determination of free aldehydes after derivatization and conjugates of aldehydes with free amino acids.

Free aldehydes in brain samples were identified by labeling with Br-tagged hydrazine probe 4-Bromo-1-ethyl-pyrazole-3-carbohydrazide (BEPC) followed by LC-MS. The resulting supernatant after protein precipitation (40 μL) was mixed with 40 μL of 20 mM BEPC and 320 μL of methanol containing 0.5% acetic acid, and then incubated at 25 °C for 2 h. Aldehyde standards (5 μM) were incubated with 5 mM BEPC in methanol containing 0.5% acetic acid at 25 °C for 2 h. The resultant incubation solution was analyzed by a Thermo Fisher Q-Exactive Ultra performance liquid chromatography Quadrupole-Orbitrap mass spectrometry (UPLC-Q/Orbitrap MS). Aldehydes were recognized by the isotopic signatures of ^79^Br/^81^Br in MS spectra, MS/MS spectra, and chromatograms. 2-Hydrazino-4-(trifluoromethyl)-pyrimidine (HTP) derivatization was further employed for aldehyde structural identification. Supernatant from striatum and SN samples (40 μL) was incubated with 10 μL of 120 μg/mL HTP and 100 μL of acetonitrile containing 0.6% acetic acid at 4 °C for 3 h. The incubation samples were prepared and analyzed by the same protocol as above. The MS/MS spectra of the aldehyde-HTP derivatives were used to further elucidate the structures of aldehydes. The conjugates of aldehydes with free amino acids were analyzed without any derivatization, using direct high-resolution MS/MS detection from the same supernatants. The proposed conjugates were identified by their high-resolution MS spectra and MS/MS spectra, which were further confirmed by the in vitro incubation of aldehyde standards with free amino acids.

### 2.3 Identification of aldehyde-protein adducts

Striatum and SN tissues underwent complete protein digestion to hydrolyze the aldehyde-protein adduct into the conjugate of an aldehyde with a single amino acid residue. The protein digestion protocol was performed according to our previously reported method [21]. In brief, striatum and SN tissues were homogenized in an ice bath with ice-cold 0.9% NaCl solution. The protein concentration of each homogenate was determined using a BCA protein assay kit. An equal amount of total protein from each sample spiked with genipin, which reacts with lysine residues of protein to generate genipin-lysine adduct, was immediately precipitated by three volumes of ice-cold methanol to obtain the protein pellet. The protein samples were washed twice with ice-cold methanol and then subjected to successive reduction and alkylation. The resultant sample solution was loaded into the 3 kDa molecular weight cutoff filter (Millipore, Darmstadt, Germany) to remove the conjugates of aldehydes with free amino acids. The protein sample was digested by incubation with protease at 37 °C for 12 h. The supernatant of digested sample was dried by N⁠_2_ blowing and redissolved by 55 μL of methanol-0.1% formic acid solution (5:95, v/v) for UPLC-Q/Orbitrap MS analysis. The aldehyde-protein adducts were identified by their high-resolution MS/MS spectra and further confirmed by comparison with the conjugates of aldehydes with free amino acids. To exclude potential ex vivo autoxidation artifacts, a parallel control experiment was performed in which tissue homogenization was carried out in a protective buffer of ice-cold 0.9% NaCl containing 1 mM desferrioxamine (DFO) and 200 μM butylated hydroxytoluene (BHT). DFO and BHT are used to inhibit spontaneous lipid peroxidation by scavenging free radicals and chelating metal ions, respectively [22], [23]. All subsequent procedures were identical to those described above. Details are provided in Supplementary Methods.

### 2.4 In vitro incubation studies

To verify the structures of conjugates of aldehydes with free amino acids, 10 μM of aldehyde standard (4-HNE, MDA, benzaldehyde, methylimidazole acetaldehyde, acrolein, lactaldehyde, or pyruvaldehyde) was incubated with 5 mM of amino acid (lysine, asparagine, or glutamine) in 100 μL phosphate-buffered saline (PBS, 0.01 M, pH 7.4) at 37 °C for 30 min. Incubation samples were then centrifuged at 12000 × g for 10 min. To assay the covalent binding behavior of acrolein and pyruvaldehyde toward a model peptide, acrolein and pyruvaldehyde at different molar concentration ratios (1:1, 0:1, and 1:0, mM) were incubated in triplicate at 37 °C for 30 min with model peptide KT5 (sequence: KYSWT) at 100 μM in PBS (0.01 M, pH 7.4) (n = 3 per group). To examine the covalent modification and aggregation of α-synuclein by aldehydes, seven aldehydes (DOPAL, 4-HNE, MDA, acrolein, pyruvaldehyde, crotonaldehyde, and malonic semialdehyde) were mixed at equimolar concentrations to prepare a stock solution. This stock was then diluted with PBS (0.01 M, pH 7.4) to yield three experimental groups, in which the recombinant rat α-synuclein (final concentration 10 μM) was incubated with the aldehyde mixture at 1:2, 1:4, and 1:8 M ratios of protein to each aldehyde. Each mixture was incubated in PBS (0.01 M, pH 7.4) at 37 °C for 4 h (n = 4 per group). Samples were analyzed by western blotting or subjected to enzymatic digestion for analysis of the conjugates of α-synuclein peptides with aldehydes.

### 2.5 Animal studies

Male Sprague-Dawley rats (200–220 g) were obtained from Hunan SJA Laboratory Animal Company (Certificate No. SCXK 2019-0004, Hunan, China) and housed in an SPF animal room (Certificate No. SYXK 2021-0003). All animals were acclimatized for 1 week. The rats were fasted with free access to water overnight before the experiments. The animal experimental procedures were approved by the Institutional Animal Use and Care Committee of Zunyi Medical University (protocol license No. ZMU21-2403-204).

The rats were randomly divided into several groups. ***Set 1***: To examine the temporal profiles of protein adduction by aldehydes, brain samples were collected at 1, 2, 4, 6, 8, 12, 24, 72, 168, and 336 h after 6-OHDA injection (n = 6–12 per time point). Prior to sample collection at 336 h (14 days), behavior testing was performed to verify the PD model. The rats received stereotaxic injection with 12 μg 6-OHDA (dissolved in 3 μL of 0.2% ascorbic acid) in the SN on the left side of the brain [24]. Rats in the sham group received 3 μL of 0.2% ascorbic acid injected into the same site. ***Set 2***: To investigate the role of aldehyde scavenging on PD, the rats were randomly divided into four groups: sham group, 6-OHDA group, 6-OHDA + Alda-1 group, and 6-OHDA + phenelzine group. Rats in 6-OHDA + Alda-1 group were administered intraperitoneally (i.p.) with 50 mg/kg Alda-1 once daily for 3 consecutive days, with the first administration performed 1 h before 6-OHDA injection. Rats in 6-OHDA + phenelzine group received i.p. injection of 15 mg/kg phenelzine once daily for 3 consecutive days, with the first administration conducted 1 h after 6-OHDA injection. Brain samples were collected at 6, 75, and 336 h after 6-OHDA injection (n = 6–12 per time point), with behavior testing performed before the final collection. ***Set 3***: To further validate protein adduction by aldehydes in PD, a rotenone-induced PD rat model of chronic mitochondrial dysfunction was established. Rats in the rotenone group received a daily subcutaneous injection of 2.5 mg/kg rotenone for 7 consecutive days, while rats in the sham group received an equal volume of vehicle via the same route. Brain samples from the SN and striatum were harvested at 0, 3, and 7 d after the final treatment (n = 6 per group per time point). These tissues were collected to analyze the levels of aldehyde-protein amino acid residue conjugates. ***Set 4***: To examine the direct toxic effects of aldehydes on dopaminergic neurons, rats received a stereotaxic injection of either individual aldehydes or aldehyde mixtures into the left SN. The aldehyde solution was infused at a rate of 0.2 μL/min, followed by a 5-min needle retention to allow gradual diffusion and minimize tissue damage. For individual injections, acrolein, 4-HNE, DOPAL, MDA, and pyruvaldehyde were each administered at 0.5 nmol in a volume of 1 μL of 0.2% ascorbic acid (n = 6 per group). For mixture injections, an aldehyde mixture comprising acrolein, 4-HNE, DOPAL, MDA, pyruvaldehyde, crotonaldehyde, and malonic semialdehyde was administered at three dose levels (0.1, 0.25, or 0.5 nmol per aldehyde) in 1 μL of 0.2% ascorbic acid (n = 12 per dose). A separate mixture containing acrolein and DOPAL was administered at 0.5 nmol per aldehyde in 1 μL of 0.2% ascorbic acid (n = 12). Brain samples were collected at 2 and 72 h after injection of aldehydes.

### 2.6 Behavioral testing

The open field test was employed to evaluate voluntary movement activity. The total distance traveled by each rat in an open field over a 10 min period was recorded. Separately, the rotarod test was used to assess motor coordination and balance. Prior to the formal test, all rats were trained on the rotarod at a fixed speed of 10 rpm. During the test, the rotation speed was increased steadily by 5 rpm every 1 min until the rat fell off the rod. The average latency to fall across three repeated trials was recorded for each animal.

### 2.7 Western blotting

Protein samples from rat brain samples were lysed in RIPA buffer. After incubation at 4 °C for 15 min, the lysates were centrifuged at 12000 × g for 30 min at 4 °C and the resulting supernatant was collected and boiled with a 5 × protein sample loading buffer. Proteins in the samples were separated by 10% or 12.5% SDS-PAGE and then transferred onto nitrocellulose membranes. The membranes were blocked with 5% nonfat milk and then subjected to immunoblotting using indicated primary antibodies, including anti-α-synuclein antibody (1:1000, ab212184, Abcam), anti-tyrosine hydroxylase (TH) antibody (1:5000, ab137869, Abcam), anti-PINK1 antibody (1:2000, 23274-1-AP, Proteintech), anti-SQSTM1 antibody (1:1000, 31403-1-AP, Proteintech), and anti-GAPDH antibody (1:10000, 60004-1-Ig, Proteintech). HRP-conjugated secondary antibodies were used for detection.

### 2.8 Immunostaining

Rat midbrain sample sections were cut at a thickness of 35 μm. Brain sections were subjected to antigen retrieval in sodium citrate buffer. For immunofluorescence staining, sections were incubated with 10% goat serum for 30 min at 37 °C, followed by overnight incubation at 4 °C with anti-TH antibody (1:250, ab137869, Abcam). Sections were then incubated with goat anti-rabbit IgG H&L (1:1000, ab150077, Abcam) at 37 °C for 45 min. For immunohistochemical staining, endogenous peroxidase activity was quenched by treating sections with 3% H_2_O_2_, followed by blocking with normal goat serum for 30 min at 37 °C. Sections were then incubated overnight at 4 °C with anti-acrolein antibody (1:100, ab240918, Abcam) or anti-Iba1 antibody (1:2000, ab178846, Abcam). After rinsing, sections were exposed to polymer peroxidase-conjugated anti-mouse/rabbit IgG for 30 min at 37 °C and the signal was visualized by diaminobenzidine. Digital images were acquired using an Olympus microscope (Olympus, Tokyo, Japan).

### 2.9 Extraction and enzymatic digestion of α-synuclein from rat brain samples

Striatum and SN tissues were homogenized with RIPA buffer. The supernatant (protein concentration, 30 μg) was boiled with a 5× protein sample loading buffer and then separated on 12.5% SDS-PAGE. Following electrophoresis, the gels were stained using the Coomassie brilliant blue staining solution. The α-synuclein protein bands in the gels were excised and recovered using a micro protein PAGE recovery kit (Solarbio, Beijing, China). The excised gel pieces were rinsed twice with water for 5 min each, crushed into smaller pieces, and then incubated with 500 μL of gel recovered solution A overnight at room temperature with gentle shaking. The resulting mixture was loaded into the gel sample filter column and centrifuged at 13000 × g for 10 min at 4 °C. The eluate was mixed with 2.5 mL of pre-cooled recovered solution B and incubated at 4 °C for 30 min. After centrifugation at 13000 × g for 15 min at 4 °C, the resultant pellet was dried under a nitrogen stream and then subjected to reduction, alkylation, and trypsin digestion, following procedures similar to those described for aldehyde-protein adduct identification. Trypsin was used to specifically cleave peptide bonds at the C-terminus side of lysine and arginine residues. The resulting peptide samples were injected into UPLC-Q/Orbitrap MS to analyze the aldehyde-modified α-synuclein peptides.

### 2.10 Analytical methods

Aldehydes and their conjugates with phenelzine, amino acids, and aldehyde-protein adducts were determined using a high-resolution UPLC-Q/Orbitrap MS system (Thermo Fisher, Waltham, MA, USA). The analytes were chromatographically separated by a Hypersil GOLD C_18_ column (150 × 2.1 mm, 1.9 μm; Thermo Fisher, Waltham, MA, USA). The mobile phase consisted of 0.1% formic acid solution (A) and methanol (B) with a flow rate of 0.3 mL/min. The gradient elution program was as follows: 25% B at 0.0-2.0 min, 25-35% B at 2.0-6.0 min, 35-58% B at 6.0-11.0 min, 58-62% B at 11.0-18.0 min, 62-65% B at 18.0-26.0 min, 65-72% B at 26.0-32.0 min, 72-85% B at 32.0-38.0 min, 85% B at 38.0-40.0 min, 85-90% B at 40.0-46.0 min, 90% B at 46.0-48.0 min, 90-25% B at 48.0-49.0 min, 25% B at 49.0-52.0 min. Data were acquired by positive electrospray ionization Full Scan-ddMS^2^ mode. Collision energy values were at 20% and 30%, respectively. The chromatogram and MS/MS data were processed by the Thermo Xcalibur 2.2 software. The analytical reliability of the semi-quantitative method for aldehyde-protein amino acid residue conjugates was validated by dilution parallelism experiments to confirm the absence of significant matrix effects. Representative striatum and SN samples were serially diluted (1:5, 1:10, and 1:20, v/v) using the initial mobile phase. The calculated relative concentrations across the dilution range were within an acceptable precision (RSD < 15%), demonstrating that the sample matrix did not significantly interfere with the ionization of the aldehyde-protein amino acid residue conjugates at the analytical concentrations used.

### 2.11 Statistical analysis

For the semi-quantitative analysis of aldehyde-protein amino acid residue conjugates, the peak area of each target conjugate was first normalized to the genipin-lysine conjugate. Then, the normalized peak area ratio of a given conjugate at 0 h was defined as 1 (or 100%), and its levels at subsequent time points were expressed as a fold change relative to its respective 0 h control. Orthogonal partial least squares-discriminant analysis (OPLS-DA) was employed to distinguish the differences of aldehyde-protein amino acid residue conjugates among several groups. Volcano plots were generated to present the differential content of aldehyde-protein amino acid residue conjugates. Comparisons between multiple groups were performed by one-way analysis of variance, followed by Tukey's post hoc test. Comparisons between two groups were performed using the two-tailed Student t-test. *P*-values < 0.05 were considered statistically significant. Data are expressed as Mean ± Standard Deviation (SD).

## 3 Results and discussion

### 3.1 Identification of free aldehydes, conjugates of aldehydes with free amino acids, and aldehyde-protein adducts in rat models of PD

The 6-OHDA-induced rat model of PD established in this study was verified by a significant reduction in both TH protein expression and the number of TH-positive neurons in the midbrain, compared with the sham group (Fig. 1A). Furthermore, the 6-OHDA-treated rats exhibited markedly decreased locomotor activity, as evidenced by reduced movement distance traveled in the open field test, as well as a significantly shortened latency to fall in the rotarod test (Fig. 1B). These data indicated that the 6-OHDA-induced rat model of PD was successfully established.

**Fig. 1:**
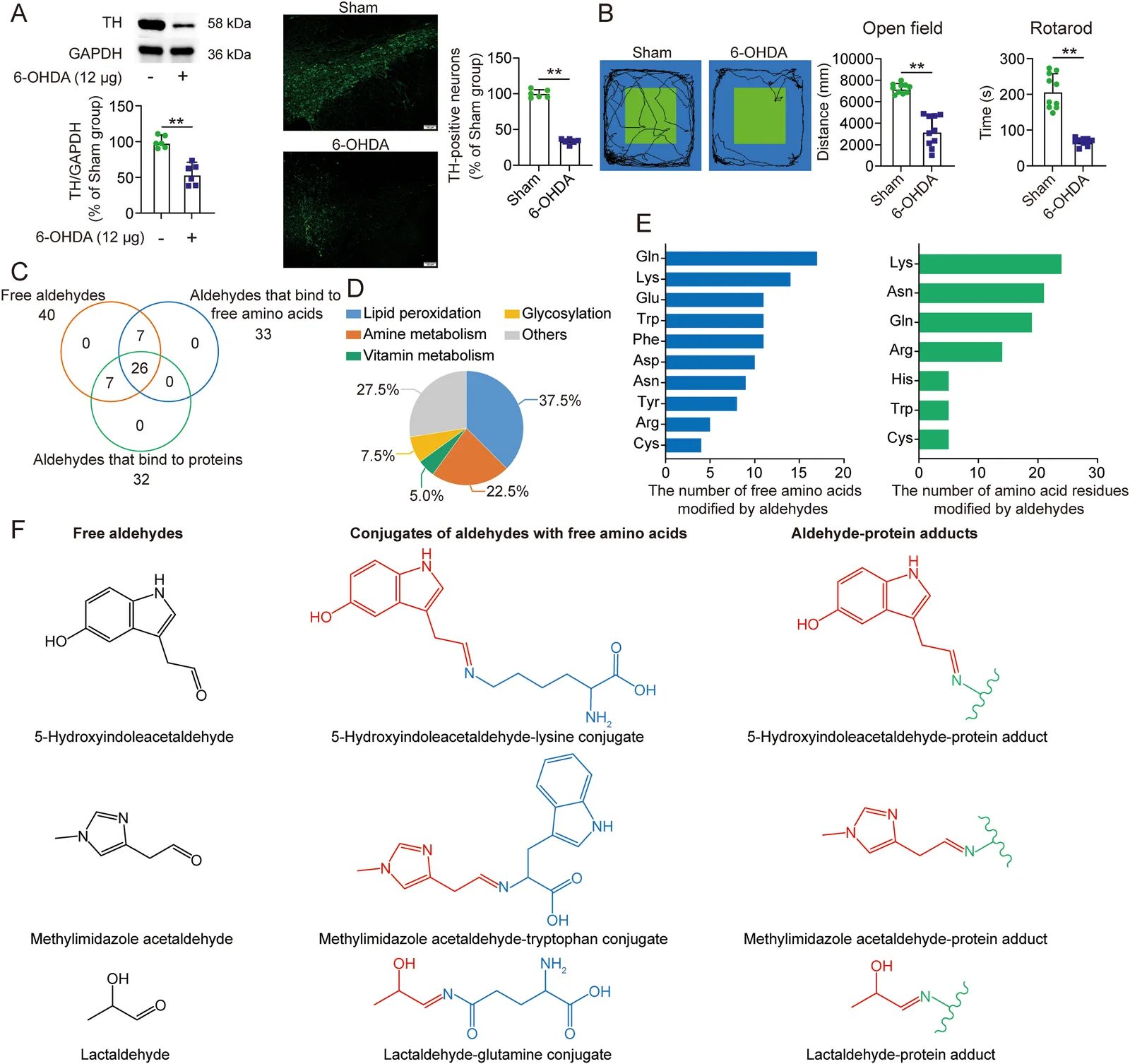
**Aldehydes found in the substantia nigra and striatum of 6-OHDA-induced rat model of PD**. (A) TH protein expression and TH-positive neurons. TH protein levels and TH-positive neurons were measured at 14 days after 6-OHDA injection using western blotting (n = 6) and immunofluorescence staining (n = 6), respectively. Scale bar = 100 μm. (B) Open-field and rotarod tests. Behavioral examinations were performed at 14 days after 6-OHDA injection (n = 10). (C) Venn diagram showing the numbers of free aldehydes, conjugates of aldehydes with free amino acids, and aldehyde–protein adducts. (D) Sources of aldehydes. (E) The number of free amino acids or protein residues modified by aldehydes. (F) Chemical structures of representative free aldehydes and their conjugates with free amino acids, and aldehyde-protein adducts. The data are presented as the mean ± SD. **P < 0.01.

Free aldehydes are inherently unstable and exert reactivity toward nucleophilic amines. Consequently, aldehydes may exist in at least three distinct forms in the rat brain: free aldehydes, conjugates of aldehydes with free amino acids, and aldehyde-protein adducts. In the present study, these three forms were identified in the SN and striatum of the 6-OHDA-induced rat model of PD. Free aldehydes in brain samples were detected via Br-tagged hydrazine labeling coupled with UPLC-Q/Orbitrap MS analysis. Aldehydes labeled with a single Br atom exhibit two isotopic precursor ions with a 1.998 Da mass difference and an intensity ratio of 100:97 in their MS spectra due to the isotopic signatures of ^79^Br/^81^Br. In chromatograms, Br-labeled aldehydes appear as peak pairs with identical retention time and similar peak areas. Correspondingly, dialdehydes labeled by two Br atoms can generate three isotopic precursor ions with 1.998 Da mass differences and a 1:2:1 intensity ratio in their MS spectra. In the chromatograms, the three isotopic precursor ions can be extracted as three ion chromatograms with identical retention time and a peak area ratio of 1:2:1. The isotopic product ion pairs were also observed in their MS/MS spectra. These distinct isotopic signatures in the MS spectra, chromatographic peak pairs, and the MS/MS spectra enable the reliable differentiation and identification of aldehydes (Fig. S1).

In this study, a total of 40 free aldehydes were identified in the SN and striatum of the 6-OHDA-induced rat model of PD (Fig. 1C). Detailed information and chemical structures of these free aldehydes are summarized in Table S1 and Fig. S2, respectively. The chemical structures of 30 aldehydes were confirmed by comparison with authentic standards, with matching retention times and MS/MS fragmentation behaviors (Fig. S3). HTP derivatization was further employed to verify the identity of free aldehydes. Thirty-two of the aldehydes found in brain samples were also identified following HTP derivatization, and their mass spectrometric characteristics and retention times were consistent with those of HTP-labeled aldehyde standards (Fig. S4). Among the 40 free aldehydes, 15 aldehydes (37.5%) were products of lipid peroxidation, such as acrolein, MDA, 4-HNE, and pentanal (Fig. 1D). Nine aldehydes (22.5%) were derived from amine oxidation, such as DOPAL, malonic semialdehyde, methylimidazole acetaldehyde, 4-hydroxyphenylacetaldehyde, and 5-hydroxyindoleacetaldehyde. The data indicated that amine metabolism constitutes a significant source of aldehydes in the SN and striatum.

Thirty-three free aldehydes were found to covalently bind to free amino acids, forming conjugates of aldehydes with free amino acids. Details of these conjugates with representative amino acids are summarized in Table S2, and the corresponding MS/MS spectra are shown in Fig. S5. The aldehydes preferentially bound to glutamine, lysine, glutamic acid, tryptophan, phenylalanine, aspartic acid, and asparagine (Fig. 1E). Protein adduction by aldehydes was assessed by characterizing the conjugates of aldehydes with individual amino acid residues of proteins after exhaustive proteolytic digestion. Thirty-two aldehydes were found to bind to the proteins. Information on these aldehyde-protein amino acid residue conjugates is listed in Table S3, and representative chromatograms and MS/MS spectra are depicted in Fig. S6. Structural identification was based on high-resolution MS/MS spectra. Notably, a single aldehyde can covalently bind to different amino acid residues within proteins. For example, 4-HNE was found to form covalent adducts with lysine, asparagine, arginine, and glutamine within proteins, yielding conjugates such as 4-HNE-lysine residue (pyrrole) conjugate and 4-HNE-asparagine residue (pyrrole) conjugate. Moreover, 4-HNE can simultaneously react with two amino acid residues to generate protein crosslinks, such as the glutamine-4-HNE-lysine crosslink (Fig. S7). Aldehyde predominantly modified lysine, asparagine, glutamine, and arginine residues, followed by histidine, cysteine, and tryptophan residues (Fig. 1E). Consistent with the findings in 6-OHDA-treated rats, these 40 free aldehydes, 33 conjugates of aldehydes with free amino acids, and 32 aldehyde-protein amino acid residue conjugates were also successfully detected in the SN and striatum of the rotenone-induced rat model of PD. Representative chemical structures of free aldehydes, conjugates of aldehydes with free amino acids, and aldehyde-protein adducts are shown in Fig. 1F. Detailed structural elucidation of aldehydes and their conjugates is exhibited in supplemental materials.

### 3.2 Temporal profile of protein adduction by aldehydes in rat models of PD

The temporal profiles of protein adduction by aldehydes in the SN and striatum of rats were determined following 6-OHDA injection. In the striatum, the levels of protein adducts formed by 7 aldehydes derived from amine oxidation increased rapidly, reaching peak levels at 6 h post-injection (Fig. 2A). These aldehydes included DOPAL, homovanillin, 4-hydroxyphenylacetaldehyde, malonic semialdehyde, 5-hydroxyindoleacetaldehyde, 4-hydroxybenzaldehyde, and methylimidazole acetaldehyde. In contrast, 6-OHDA administration did not elevate the extent of protein adduction by benzaldehyde and 2-phenylpropanal. The levels of protein adducts formed by 10 aldehydes derived from lipid peroxidation also exhibited a rapid increase, peaking at approximately 4 h, such as 4-HNE, MDA, and acrolein (Fig. 2B), whereas the extent of protein adduction by 2-nonenal, butanal, and 2-decenal remained relatively constant throughout the time course. The remaining protein adducts formed by the other 10 aldehydes mostly reached maximum levels between 4 and 6 h (Fig. 2C). The temporal profiling of protein adduction by aldehydes in the SN was generally similar to those observed in the striatum (Fig. 2D–F). Overall, the levels of 27 aldehyde-protein adducts in the SN and striatum were increased by up to approximately 5-fold relative to the 0 h baseline within 6 h following 6-OHDA injection (Table S4). In the chronic rotenone model, of the 27 aldehyde–protein adducts identified above, the levels of aldehyde-protein adducts exhibited a progressive trend, beginning with a modest elevation of 16 species at 3 days and expanding to 24 species with an increase of up to approximately 3-fold by 7 days (Tables S5 and S6). A parallel control experiment using a protective homogenization buffer yielded comparable elevations in aldehyde–protein adducts at 4 and 6 h compared with routine homogenization (*P* > 0.05, Fig. S8), confirming that the rapid elevation of aldehyde-protein adducts after 6-OHDA injection reflects changes occurring in vivo rather than ex vivo autoxidation artifacts. Taken together, these data suggest that protein adduction by a diverse array of aldehydes is a conserved feature across various PD models.

**Fig. 2:**
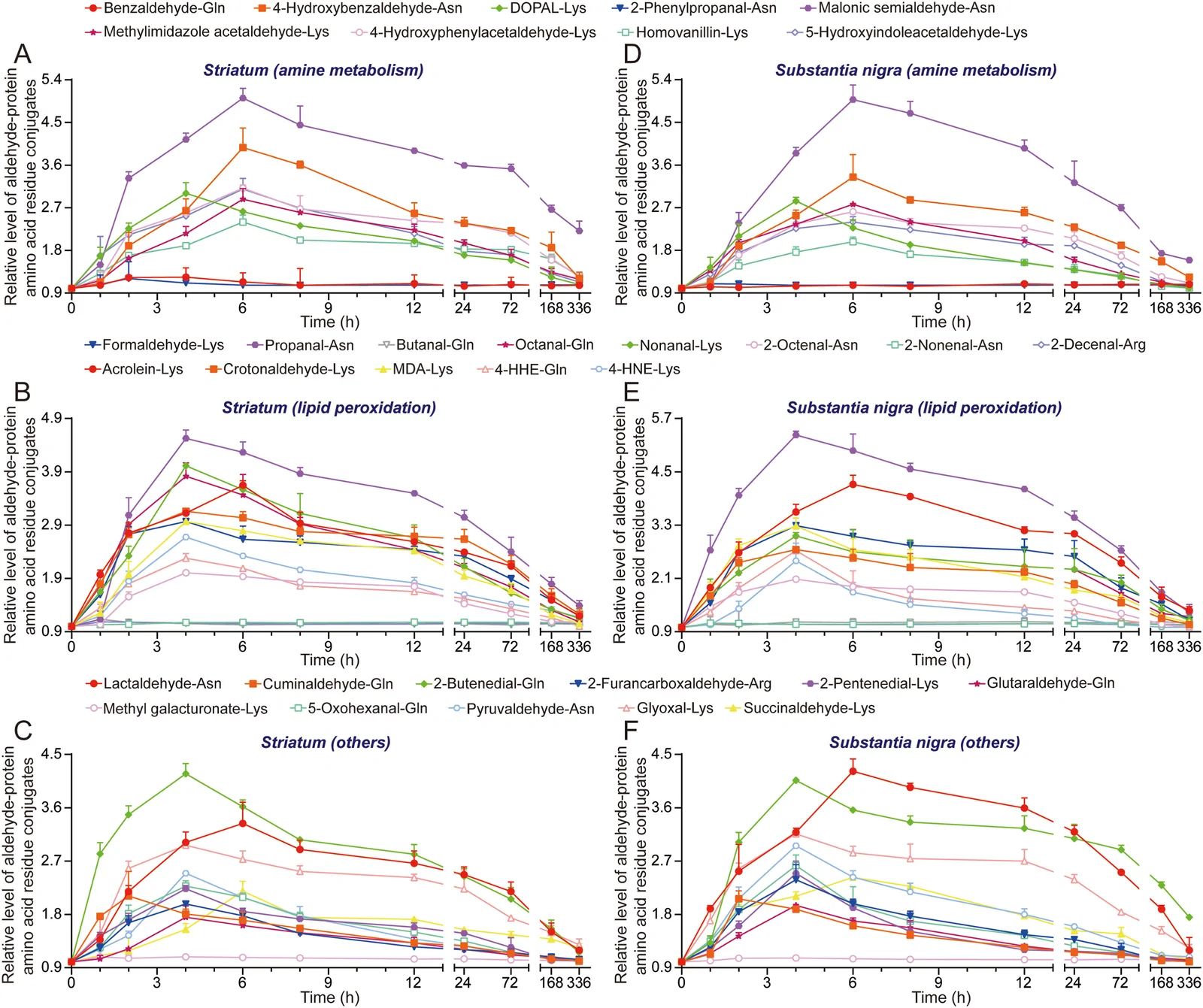
**Temporal profiles of protein adduction by aldehydes in the 6-OHDA-induced rat model of PD.** Protein adduction by aldehydes derived from (A) amine metabolism, (B) lipid peroxidation, and (C) other sources in the striatum. Protein adduction by aldehydes derived from (D) amine metabolism, (E) lipid peroxidation, and (F) other sources in the substantia nigra. The aldehyde-protein amino acid residue conjugates were measured at sham-operated control (0 h) and at 1, 2, 4, 6, 8, 12, 24, 72, 168, and 336 h after 6-OHDA injection (n = 6 per time point). The relative abundance of each aldehyde-protein amino acid residue conjugate at 0 h was normalized to 1. The data are presented as the mean ± SD.

### 3.3 Effects of Alda-1 and phenelzine on protein adduction by aldehydes and dopaminergic neuron loss in the 6-OHDA-induced rat model of PD

Alda-1 is a highly selective activator of aldehyde dehydrogenase-2 (ALDH2), an enzyme that catalyzes the oxidation of aldehydes [25]. Phenelzine contains a hydrazine group that enables it to trap reactive aldehydes. In this study, Alda-1 and phenelzine were employed to modulate aldehyde levels and to evaluate the role of aldehydes in 6-OHDA-induced dopaminergic neuron loss. As shown in Fig. 3A, the clear separation of four experimental groups in the scores plots analyzed by an OPLS-DA model indicated distinct differences in the levels of aldehyde-protein adducts in the SN samples among the sham, 6-OHDA, 6-OHDA + Alda-1, and 6-OHDA + phenelzine groups. Volcano plots showed the significantly altered aldehyde-protein adducts (log_2_ |fold change| ≥ 1.2 and *P* < 0.05) between the sham and 6-OHDA groups. Administration of either Alda-1 or phenelzine caused significant changes in aldehyde-protein adducts in 6-OHDA-treated rats (Fig. 3B). The fold changes and corresponding *P* values of protein adducts between the different groups are shown in Table S4, Tables S7 and S8.

**Fig. 3:**
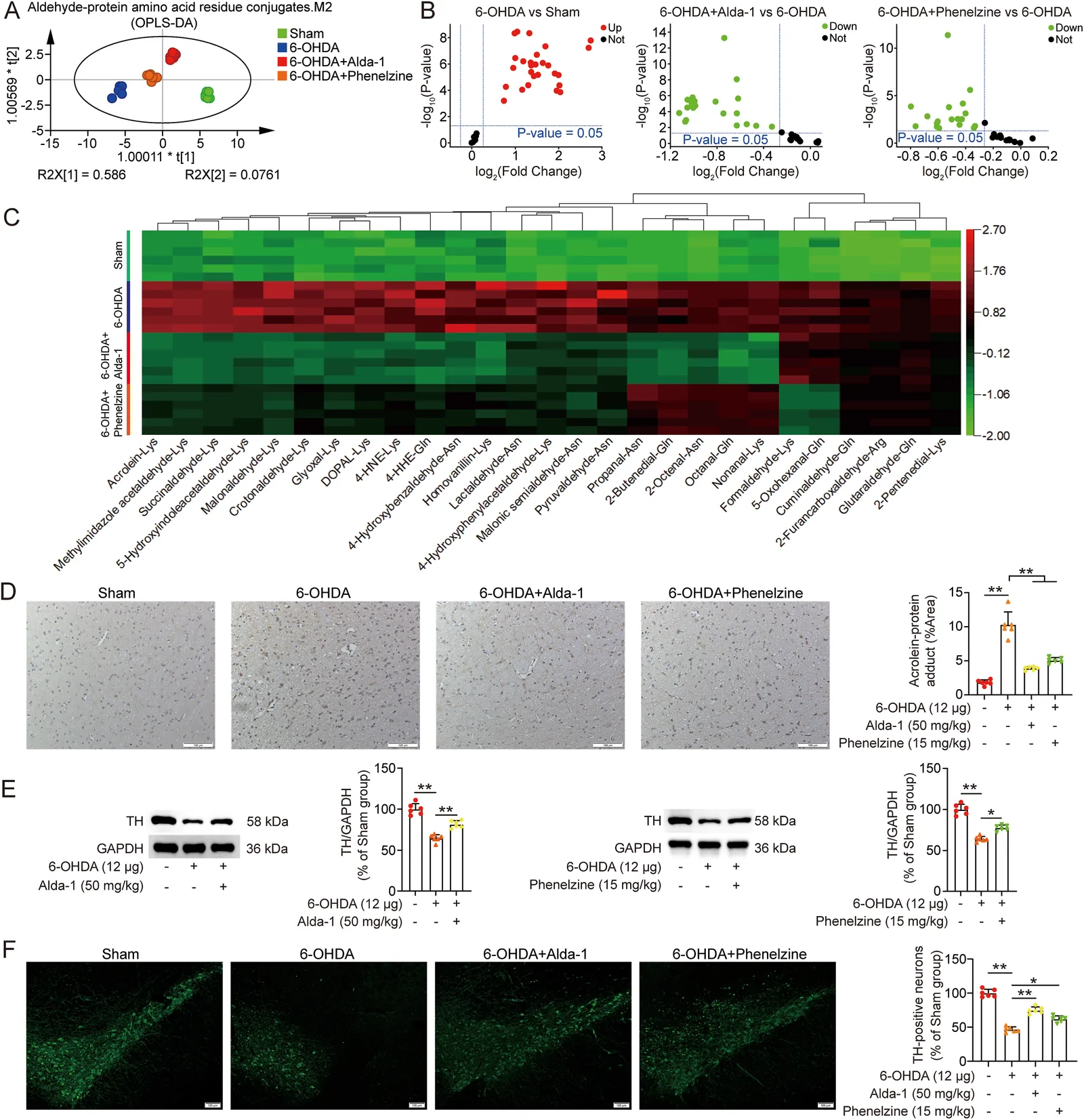
**Effects of Alda-1 or phenelzine on protein adduction by aldehydes and dopaminergic neuron loss in the 6-OHDA-induced rat model of PD.** (A) Orthogonal partial least squares discriminant analysis (OPLS-DA) score plot of aldehyde-protein adducts in the substantia nigra of the sham, 6-OHDA, 6-OHDA + Alda-1, and 6-OHDA + phenelzine groups (n = 6). (B) Volcano map of aldehyde-protein adducts in the substantia nigra (sham versus 6-OHDA, 6-OHDA + Alda-1 versus 6-OHDA, and 6-OHDA + phenelzine versus 6-OHDA) (n = 6). The fold change is displayed on the X-axis, and the significance of changes (−log_10_ P-value) is shown on the Y-axis. Each symbol represents a distinct aldehyde-protein amino acid residue conjugate. (C) Heat map of aldehyde-protein adducts in the substantia nigra of sham, 6-OHDA, 6-OHDA + Alda-1, and 6-OHDA + phenelzine group (n = 6). Red represents high levels of protein adducts, whereas green indicates lower levels. (D) Acrolein-protein adducts determined by immunohistochemical staining (n = 6), Scale bar = 100 μm. (E) TH protein levels in the substantia nigra measured by western blotting (n = 6). (F) TH-positive neurons in the midbrain determined by immunofluorescence staining (n = 6), Scale bars = 100 μm. Rats in the 6-OHDA + Alda-1 group received 50 mg/kg Alda-1 (i.p.) once daily for three consecutive days, with the first dose administered 1 h prior to 6-OHDA injection, whereas rats in the 6-OHDA + phenelzine group were given 15 mg/kg phenelzine (i.p.) once daily for three consecutive days, beginning 1 h after 6-OHDA injection. The TH protein expression, TH-positive neuron number, and acrolein-protein adduction were assayed at 75 h after 6-OHDA injection. The data are presented as the mean ± SD. *P < 0.05, **P < 0.01.

The alterations in aldehyde-protein adducts across four experimental groups were further depicted in the heat map (Fig. 3C). The levels of 27 aldehyde-protein adducts were increased in the SN of the 6-OHDA group, compared with the sham group. Treatment with Alda-1 significantly reduced the levels of amino acid residue conjugates with 21 aldehydes in 6-OHDA-treated rats. Similarly, the elevated extent of protein adduction involving 18 aldehydes in 6-OHDA-treated rats was significantly attenuated following phenelzine administration. Representative images showed that the elevated acrolein-protein adduction-positive area induced by 6-OHDA was significantly reduced after treatment with Alda-1 or phenelzine (Fig. 3D). Notably, the phenelzine-aldehyde conjugates were detected in the SN of 6-OHDA-treated rats that received phenelzine (Fig. S9), but not in those without the drug, thereby providing direct evidence for the aldehyde-scavenging activity of phenelzine in the brain. Moreover, treatment with either Alda-1 or phenelzine significantly attenuated the reduction in TH protein expression and the loss of TH-positive neurons in 6-OHDA-treated rats (Fig. 3E and F), which were consistent with the observed changes in the protein adduction by aldehydes. The behavioral tests confirmed that Alda-1 and phenelzine improved the motor performance of 6-OHDA-treated rats (Fig. S10). These data suggest that covalent modification of proteins by multiple aldehydes in the SN and striatum plays an important role in 6-OHDA-induced dopaminergic neuronal damage.

### 3.4 Direct toxic effects of endogenous aldehydes on dopaminergic neurons

The direct toxic effects of aldehydes on dopaminergic neurons were first assessed in rats following separate stereotaxic injection of individual aldehydes into the left SN of rats. Compared with the sham group, individual aldehydes at 0.5 nmol did not significantly reduce TH protein expression (Fig. 4A). To investigate whether exposure to multiple aldehydes exerts additive neurotoxicity, an equimolar mixture of aldehydes (acrolein, 4-HNE, DOPAL, MDA, pyruvaldehyde, crotonaldehyde, and malonic semialdehyde) was injected at 0.1, 0.25, or 0.5 nmol per aldehyde. As shown in Fig. 4B and C, the aldehyde mixture at 0.5 nmol significantly reduced TH protein expression and the number of TH-positive neurons in the SN. At 0.25 nmol per aldehyde, the mixture caused a mild reduction, whereas the 0.1 nmol per aldehyde dose had no significant effect. In addition, the aldehyde mixture at 0.5 nmol induced microglial activation in the SN (Fig. S11). Separately, it also elevated the protein levels of PTEN-induced putative kinase 1 (PINK1) and sequestosome 1 (SQSTM1) in the SN, and reduced ATP levels in the striatum (Fig. S12), indicating aldehyde-induced mitochondrial dysfunction accompanied by alterations in mitophagy-related markers. By contrast, a two-aldehyde mixture (acrolein and DOPAL) at 0.5 nmol did not significantly alter TH protein expression or the number of TH-positive neurons. Heat map analysis of aldehyde–protein amino acid residue conjugates in the SN revealed that the aldehyde mixture at 0.5 nmol markedly elevated the levels of multiple aldehyde–protein adducts (Fig. 4D). These findings indicate that endogenous aldehydes can directly induce dopaminergic neuron loss.

**Fig. 4:**
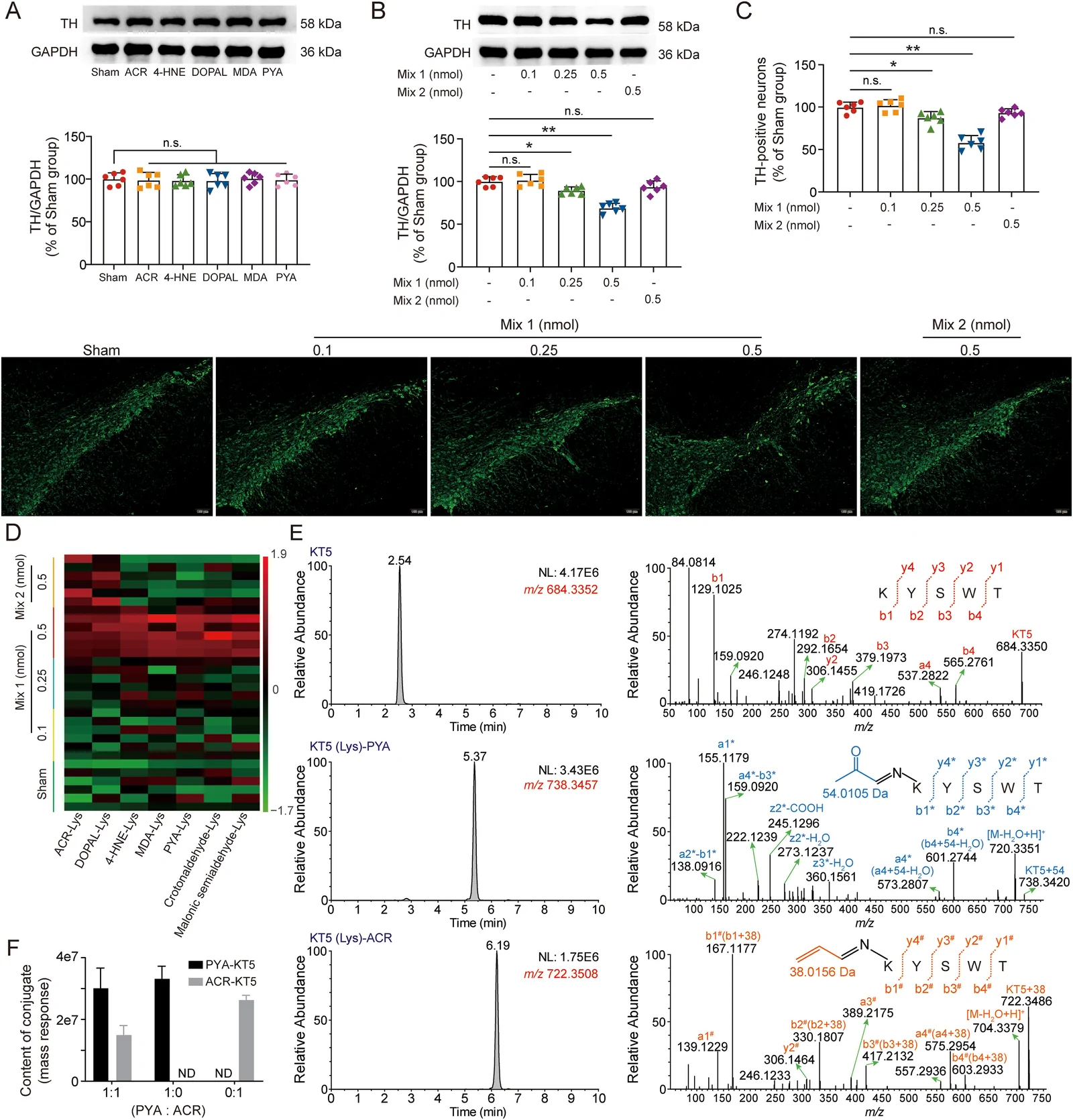
**Direct toxic effects of aldehydes on dopaminergic neurons**. (A) TH protein levels in the substantia nigra measured by western blotting at 72 h after separate injection of individual aldehydes (0.5 nmol) into the left substantia nigra of rats (n = 6 per group). (B) TH protein levels measured by western blotting and (C) TH-positive neurons detected by immunofluorescence staining at 72 h after injection of the aldehyde mixture into the left substantia nigra of rats (n = 6 per group). Mix 1 contained acrolein (ACR), 4-HNE, DOPAL, MDA, pyruvaldehyde (PYA), crotonaldehyde, and malonic semialdehyde at three dose levels (0.1, 0.25, or 0.5 nmol per aldehyde). Mix 2 contained ACR and DOPAL at 0.5 nmol per aldehyde. Scale bar = 100 μm. (D) Heat map of aldehyde-protein adducts at 2 h after injection of aldehydes. (E) Extracted ion chromatogram and MS/MS spectra of the model peptide KT5 and aldehyde-modified KT5 peptides. (F) Levels of aldehyde-peptide KT5 conjugates in incubation. ACR and PYA at different concentrations (1:1, 0:1, and 1:0, mM) were incubated with the model peptide KT5 (100 μM) in PBS at 37 °C for 30 min (n = 3). The data are presented as the mean ± SD. *P < 0.05, **P < 0.01. n.s. represents not significant. ND represents not detected.

We next examined the covalent binding behavior of acrolein and pyruvaldehyde toward a model lysine-containing peptide (KT5, amino acid sequence: KYSWT) in PBS. Acrolein and pyruvaldehyde were capable of modifying the lysine residue of the model peptide (Fig. 4E). Peaks with retention times of 5.37 min and 6.19 min in the extracted ion chromatograms of *m*/*z* 738.3457 and 722.3508 were assigned as KT5(Lys)-pyruvaldehyde and KT5(Lys)-acrolein, respectively. The protonated mass of these conjugates was 54.0105 and 38.0156 Da greater than that of unmodified KT5 (*m*/*z* 684.3350). Corresponding mass shifts of +54 Da and +38 Da were also observed in the b-type fragment ions in the MS/MS spectra of the respective conjugates. When incubated with equimolar concentrations of both aldehydes, the yield of the KT5-pyruvaldehyde conjugate was 1.8-fold higher than that of KT5-acrolein conjugate (Fig. 4F). Moreover, the level of KT5-acrolein conjugate formed in the single-aldehyde incubation was much higher than that observed in the mixed-aldehyde incubation. The data suggest that different aldehydes can simultaneously modify the same amino acid residue of proteins and that pyruvaldehyde exhibits greater reactivity toward the model peptide than acrolein. Collectively, the data indicate that the “additive effects” of covalent protein modification by multiple aldehydes may contribute to dopaminergic neuron loss.

### 3.5 Covalent modification and aggregation of α-synuclein by aldehydes

One pathological hallmark of PD is the accumulation of aggregated α-synuclein as Lewy bodies within surviving SN neurons. In the present study, α-synuclein isolated from 6-OHDA-treated rats was found to be covalently modified by 12 aldehydes. Following selective proteolysis at lysine residues, twelve peptide fragments were generated and analyzed, among which five were found to be covalently modified by aldehydes (Fig. 5A). The peptide fragment GLSK was identified as a major target, with its lysine residue modified by eight aldehydes, predominantly products of amine oxidation, including DOPAL, pyruvaldehyde, malonic semialdehyde, 4-hydroxybenzaldehyde, methylimidazole acetaldehyde, 5-hydroxyindoleacetaldehyde, 4-hydroxyphenylacetaldehyde and MDA (product of lipid peroxidation). The peptide fragment AK was modified by crotonaldehyde, 4-HNE, MDA, and pyruvaldehyde. The peptide fragments QGVAEAAGK and DQMGK were modified by propanal and octanal, respectively. Detailed information on the α-synuclein peptide fragments modified by aldehydes is listed in Table S9. Representative chromatograms and MS/MS spectra of pyruvaldehyde-modified GLSK fragment are shown in Fig. 5B, with full structural elucidation exhibited in supplementary materials. Furthermore, the GLSK-pyruvaldehyde conjugate generated from in vitro incubation of pyruvaldehyde with recombinant rat α-synuclein exhibited retention time and fragmentation pattern identical to those observed in rat brain samples, providing strong evidence for covalent modification of the GLSK peptide by pyruvaldehyde.

**Fig. 5:**
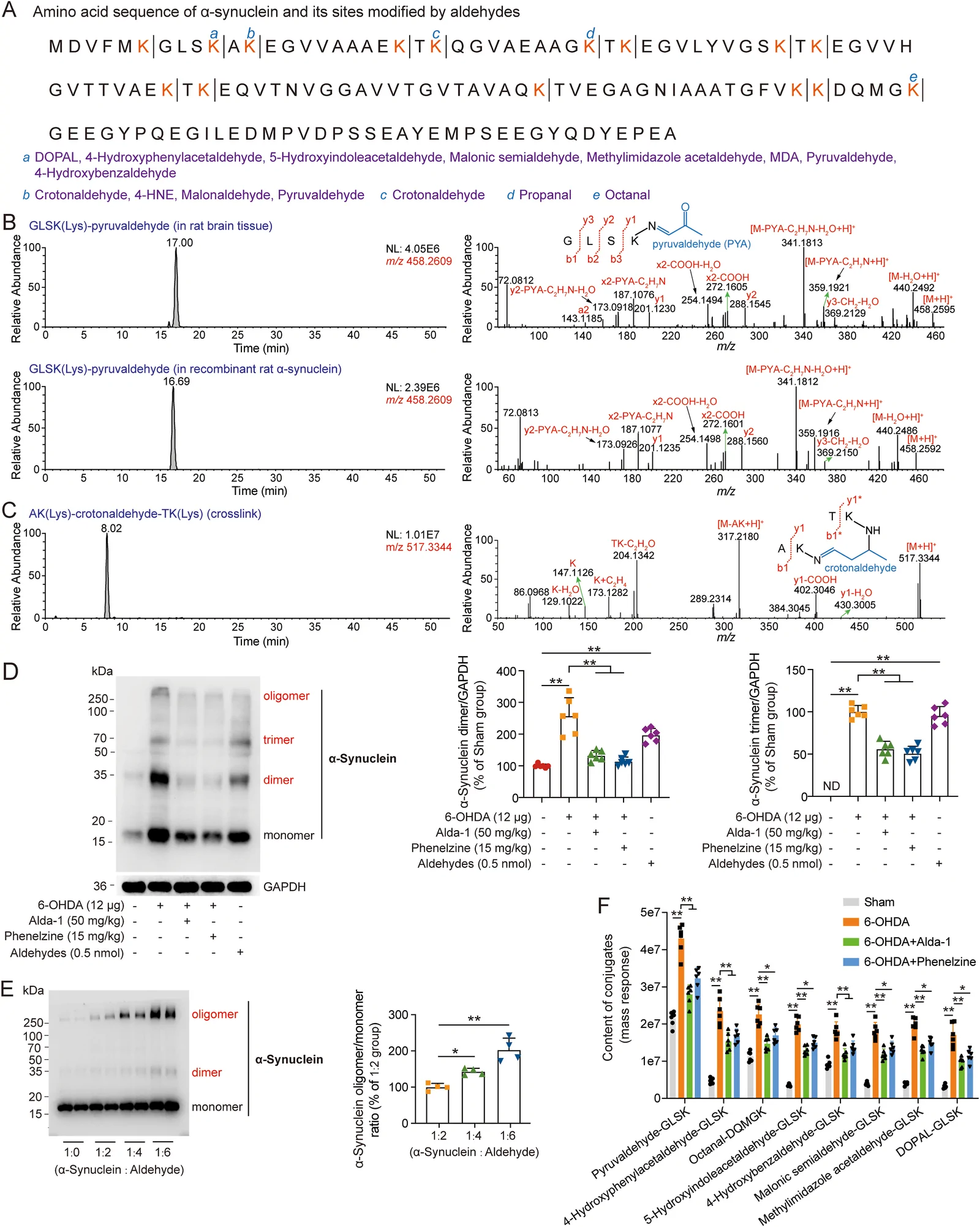
**Covalent modification and aggregation of α-synuclein by aldehydes.** (A) Amino acid sequence of rat α-synuclein and its modification sites by aldehydes in the 6-OHDA-induced rat model of PD. α-Synuclein was extracted from brain at 6 h after 6-OHDA injection. (B) Extracted ion chromatograms and MS/MS spectra of the pyruvaldehyde-modified GLSK peptide in the 6-OHDA-treated rats and following in vitro incubation of pyruvaldehyde with recombinant rat α-synuclein protein. (C) Extracted ion chromatograms and MS/MS spectra of the AK-crotonaldehyde-TK crosslink in brain samples from 6-OHDA-treated rats. (D) Aggregation of α-synuclein in rats. Rats received a single injection of 6-OHDA, followed by either no treatment or once-daily i.p. injections of 50 mg/kg Alda-1 (first dose given 1 h before 6-OHDA) or 15 mg/kg phenelzine (first dose given 1 h after 6-OHDA) for three consecutive days. The other group rats received a single injection of the equimolar aldehyde mixture consisting of acrolein, 4-HNE, DOPAL, MDA, crotonaldehyde, pyruvaldehyde and malonic semialdehyde at 0.5 nmol each. The protein expression of α-synuclein was determined at 75 h and 72 h after injection of 6-OHDA and aldehydes, respectively (n = 6). (E) Aggregation of α-synuclein induced by aldehydes in vitro. Recombinant rat α-synuclein was incubated with the same aldehyde mixture at protein-to-aldehyde molar ratios of 1:2, 1:4, and 1:6 (α-synuclein: each aldehyde) in PBS at 37 °C for 4 h (n = 4). (F) Levels of aldehyde-modified α-synuclein peptide fragments in rats. Rats were treated with 6-OHDA, 6-OHDA + Alda-1, or 6-OHDA + phenelzine. The aldehyde-modified GLSK peptides were determined at 75 h after 6-OHDA injection (n = 6). The data are presented as the mean ± SD. *P < 0.05, **P < 0.01. ND represents not detected.

Importantly, a crotonaldehyde-derived crosslink between AK and TK peptides and a 4-HNE-derived AK-AK crosslink were observed in brain samples from 6-OHDA-treated rats. The structure of the AK-crotonaldehyde-TK crosslink was supported by several diagnostic fragment ions (Fig. 5C). Product ions at *m*/*z* 430.3005 and 402.3046 corresponded to the loss of H_2_O and HCOOH after y_1_ cleavage, respectively. The base peak at *m*/*z* 317.2180 was generated from the loss of the AK peptide moiety, while the ion at *m*/*z* 204.1342 originated from the TK peptide after the loss of the C_2_H_2_O moiety from threonine. Additional product ions at *m*/*z* 147.1126, 129.1022, and 86.0986 were attributed to fragments of the lysine residue. The structural elucidation and MS/MS spectra of the AK-4-HNE-AK crosslink are exhibited in Fig. S13. Aldehyde-mediated crosslinking of α-synuclein peptides may present a key mechanism underlying α-synuclein aggregation.

Stereotaxic injection of 6-OHDA into rats induced the formation of α-synuclein aggregates, including dimers, trimers, and high-molecular-weight oligomers, and this aggregation was significantly ameliorated by treatment with either Alda-1 or phenelzine (Fig. 5D). Injection of the aldehyde mixture into rats also caused a significant increase in α-synuclein oligomerization. In vitro, incubation of recombinant rat α-synuclein with this aldehyde mixture induced α-synuclein aggregation in a concentration-dependent manner (Fig. 5E). Additionally, treatment with Alda-1 or phenelzine significantly reduced the levels of aldehyde-modified α-synuclein peptide fragments in 6-OHDA-treated rats (Fig. 5F). Taken together, these results demonstrate that covalent modification by multiple aldehydes promotes α-synuclein aggregation in the 6-OHDA-induced rat model of PD.

## 4 Discussion

In the present study, 40 distinct free aldehydes were identified in the SN and striatum of both 6-OHDA- and rotenone-induced rat models of PD. Beyond classical lipid peroxidation, amine oxidation emerged as a primary source of these reactive aldehydes. Crucially, we mapped the covalent lysine modification of α-synuclein by 12 different aldehydes, which facilitated peptide crosslinking and subsequent α-synuclein aggregation. Furthermore, we identified an additive effect of covalent modification by multiple aldehydes, which collectively causes neuron death. Taken together, these findings suggest that protein adduction by multiple aldehydes contributes to α-synuclein aggregation and dopaminergic neuron loss in rat models of PD (Scheme 1).

**Scheme 1:**
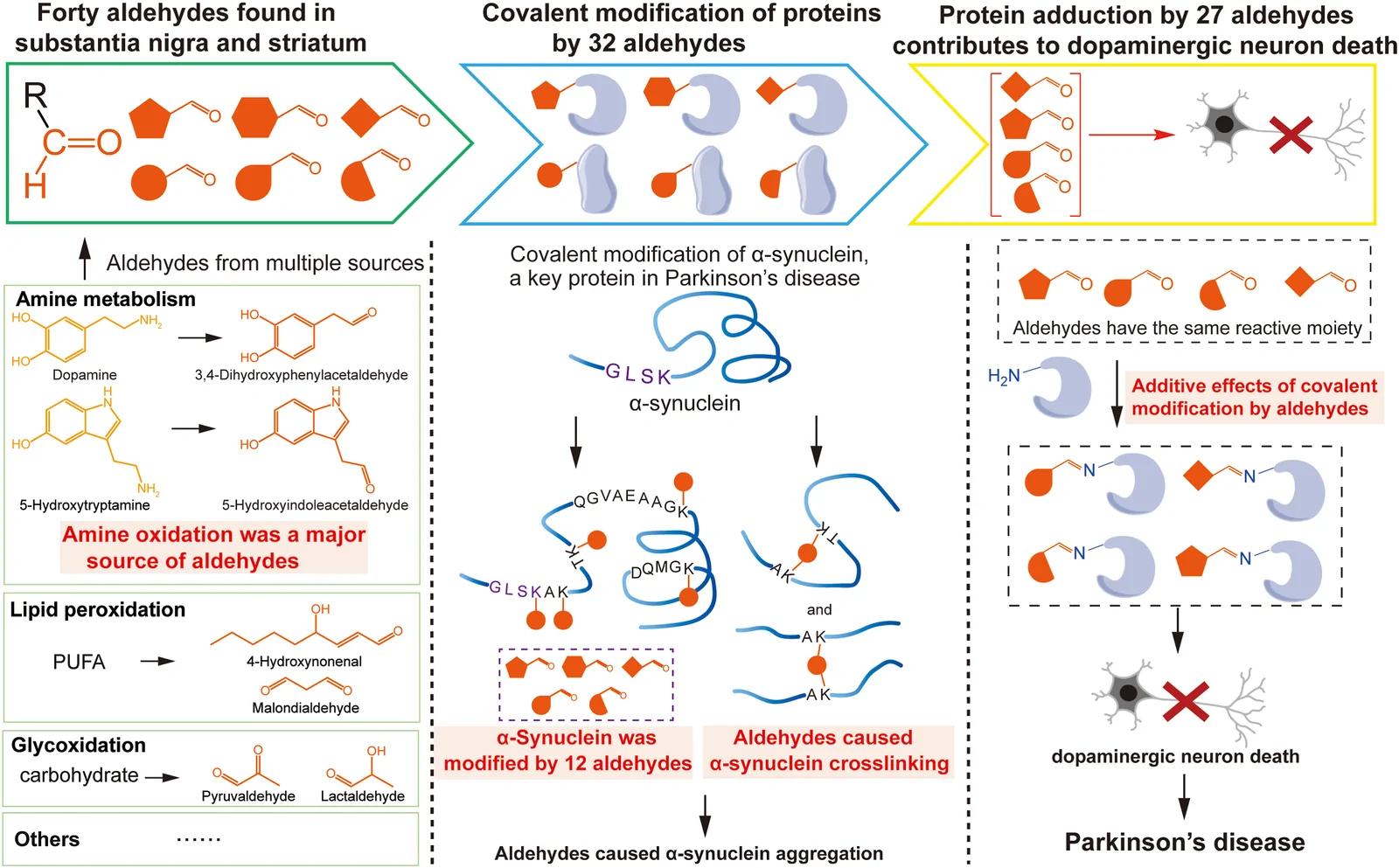
Graphic illustration of the role of protein adduction by multiple aldehydes in α-synuclein aggregation and dopaminergic neuron loss in Parkinson's disease.

The reliable identification of endogenous aldehydes remains inherently challenging due to their reactivity and short half-life. In particular, brain homogenates are susceptible to ex vivo autoxidation, which can falsely generate artifactual aldehydes upon exposure to atmospheric oxygen, trace free iron, or endogenous reducing agents such as ascorbic acid [22]. To ensure that the identified aldehyde profiles accurately reflect true endogenous aldehydes rather than post-mortem processing artifacts, we implemented several rigorous stabilization and validation strategies. These included liquid nitrogen preservation of tissue samples, strict temperature control (ice-cold conditions) and rapid handling during the entire pre-analytical process. In brief, the collected brain tissues were promptly dissected on ice to isolate the SN and striatum, immediately snap-frozen in liquid nitrogen, and subsequently homogenized for no more than 5 min. The homogenate was immediately precipitated with ice-cold methanol, and subsequently the supernatant and the protein pellet were maintained at 4 °C. For the identification of free aldehydes, we employed a Br-tagged hydrazine (BEPC) labeling approach to facilitate the rapid stabilization of aldehydes and to utilize the unique isotopic signature of Br for robust identification. Derivatization conditions were optimized to ensure rapid and complete conversion of free aldehydes to stable derivatives. Furthermore, the structural identification of these aldehydes was cross-validated using a second hydrazine probe, HTP. This dual-probe approach minimizes the possibility of misidentifying random oxidation products as endogenous aldehydes. For the analysis of aldehyde–protein adducts, the protein pellet was washed twice with ice-cold methanol to remove residual lipids and reduce the risk of autoxidation during subsequent enzymatic digestion. Ultrafiltration was employed to selectively retain covalently modified proteins. The parallel control homogenization experiment employing a protective buffer further supports the specificity and reliability of the aldehyde–protein adduct measurements.

We successfully identified 40 free aldehydes in brain samples of two distinct PD models induced by 6-OHDA and rotenone. To the best of our knowledge, 22 of these aldehydes are reported in the rat brain for the first time. The remaining eighteen aldehydes have been previously reported in the brain, such as DOPAL, pyridoxal, glyoxal, pyruvaldehyde, and several lipid peroxidation products (4-HNE, MDA, acrolein, etc.) [10], [26], [27]. Crucially, our data reveal that the aldehyde species in the PD rat brain are far more complex than previously recognized. Specifically, 15 identified aldehydes were products of lipid peroxidation, including well-known species such as 4-HNE, MDA, and acrolein [6]. Nine aldehydes were derived from amine oxidation, such as DOPAL (the intermediate of dopamine oxidation) [15], 5-hydroxyindoleacetaldehyde (the intermediate metabolite of 5-hydroxytryptamine) [28], and 4-hydroxyphenylacetaldehyde, malonic semialdehyde, and methylimidazole acetaldehyde likely originating from tyramine, alanine, and histamine [29], [30], [31]. Additionally, we identified glyoxal and pyruvaldehyde from glucose oxidation, lactaldehyde from lactic acid [32], pyridoxal and pyridoxal 5′-phosphate from vitamin metabolism. These diverse origins suggest that aldehyde overload in the PD brain arises from multiple biochemical pathways beyond simple oxidative stress. Following the characterization of free aldehydes, we systematically investigated their conjugates with free amino acids and protein adducts, guided by the chemical structures of free aldehydes and amino acids, established conjugation reaction (Michael addition or Schiff base), and previously reported aldehyde-protein amino acid residue conjugates [33]. The simultaneous discovery of three distinct forms of aldehydes indicated the high accuracy of our structure identification. These conjugates of aldehydes with different amino acid residues also supported the identification of protein adduction by aldehydes. These aldehyde-protein amino acid residue conjugates, characterized by their high stability and long half-life, served as reliable chemical markers for quantifying protein adduction by aldehydes. Importantly, the extensive detection of these adducts provides definitive biological evidence that reactive aldehydes persist in the PD brain at concentrations sufficient to bypass endogenous scavengers (e.g., GSH), enabling them to covalently modify functional proteins and drive dopaminergic neurodegeneration.

6-OHDA is a classic neurotoxin that triggers rapid reactive oxygen species (ROS) production and selectively induces lesions in the nigrostriatal dopaminergic system [34]. Given that oxidative stress is a fundamental hallmark shared across diverse PD etiologies, the 6-OHDA-induced acute model remains a highly relevant and widely utilized tool for investigating early molecular triggers. In the present study, our temporal profiling data revealed that protein adduction by multiple aldehydes occurred within 4–6 h following 6-OHDA injection, significantly preceding the onset of detectable dopaminergic neuron degeneration. This critical temporal gap suggests that aldehyde-mediated covalent modification is an early, potentially causative event in the pathogenic cascade. To validate the universality of this mechanism, we further employed the rotenone-induced chronic model, which mimics progressive mitochondrial dysfunction. Despite their contrasting temporal dynamics, a core signature of early, largely overlapping, and elevated aldehyde-protein adducts was consistently identified. This cross-model verification underscores that aldehyde overload may be an initial and conserved pathological hallmark regardless of PD pathology. Crucially, our data reveal that, within the elevated aldehyde-protein adducts, these reactive aldehydes originate not only from classical lipid peroxidation but primarily (approximately 60%) from abnormal amine oxidation, as evidenced by the identification of 16 previously unreported aldehydes derived from amine oxidation in the rat brain, such as 5-hydroxyindoleacetaldehyde, malonic semialdehyde, methylimidazole acetaldehyde, 4-hydroxyphenylacetaldehyde, homovanillin, and lactaldehyde. By extending the pathological spectrum beyond traditionally studied markers like DOPAL and acrolein, these findings establish that a much broader spectrum of aldehydes may participate in dopaminergic neuron loss in PD.

The aggregation of α-synuclein is a crucial mechanism underlying dopaminergic neurodegeneration. α-Synuclein is particularly susceptible to covalent modification due to its lysine-rich sequence (15 residues out of 140), high local concentration at the presynaptic terminals (around 40 μM), and intrinsically unfolded state that leaves lysine residues highly exposed [10]. The disordered state of α-synuclein is largely maintained by the positive charges of its lysine residues, which provide electrostatic repulsion to prevent intramolecular interactions and β-sheet formation. Aldehydes are hard electrophiles that readily react with the ε-amino group of lysine residues, the hard nucleophile. Previous studies have demonstrated that α-synuclein is covalently modified by several well-known aldehydes from lipid peroxidation and glycation in PD patients, animal models and in vitro studies, including DOPAL, 4-HNE, MDA, acrolein, pyruvaldehyde, and glyoxal, as determined by immunohistochemistry and biochemical methods [11], [12], [13], [14]. Such covalent modification can alter the local electrostatic surface potential and introduce spatial constraints that disrupt the native repulsion, driving a conformational transition of α-synuclein from α-helix/random coil to a β-sheet-rich structure that favors oligomerization [35], [36], [37]. Here, our study further reveals that α-synuclein in 6-OHDA-treated rats underwent covalent modification by a broader array of 12 distinct aldehydes than previously recognized, with amine oxidation being a predominant source. The lysine residues within our identified peptide fragments, including K10 (within the GLSK fragment, the major target for aldehyde modification), K12 (within the AK fragment), K23 (within the TK fragment), and K32 (within the QGVAEAAGK fragment), are located within the N-terminal domain responsible for forming the α-helix structure that facilitates binding to lipid membranes. Therefore, multiple aldehyde-mediated covalent modifications of the lysine residues in α-synuclein may drive the disorder-to-β-sheet transition and shift the structural equilibrium toward the unbound, aggregation-prone form.

In addition to the single-residue modifications, aldehyde-induced crosslinks have been reported to lock α-synuclein into stable oligomeric conformations [38]. In this study, we further characterized two novel covalent crosslinks in α-synuclein: AK-crotonaldehyde-TK and AK-4-HNE-AK, both located within the N-terminal peptide region that actively participates in β-sheet propagation with the NACore region during aggregation [39]. Thus, the spatial constraints introduced by these crosslinks are highly likely to disrupt the native disordered conformation of α-synuclein, enhancing its aggregation propensity in vivo. Our in vivo and in vitro experiments further confirmed that aldehydes directly induced α-synuclein aggregation. Beyond structural changes, aldehyde modification at these specific residues may carry functional consequences. The modified lysine residues K10, K12, K23, and K32 are not only essential for lipid membrane binding, but also serve as primary targets for ubiquitination [40]. Additionally, K10 is a pivotal residue mediating extracellular proteolysis and clearance. Thus, their covalent modification by aldehydes may simultaneously impair proteasome-dependent degradation and extracellular clearance of α-synuclein, collectively promoting α-synuclein accumulation and aggregation.

ALDH2 plays a central role in the metabolic clearance of aldehydes, including neurotoxic DOPAL, acrolein, and 4-HNE. ALDH2 activity is decreased in the SN of PD models [41]. Loss of ALDH2 activity is recognized as a key pathogenic mechanism underlying PD [16]. Conversely, activation of ALDH2 can ameliorate dopaminergic neuron death in a rat model of PD [42]. Accordingly, we employed Alda-1, a highly selective small-molecule activator of ALDH2, to promote metabolic clearance of aldehydes in the 6-OHDA-induced PD model. To further verify the contribution of aldehyde clearance, rather than other actions of ALDH2 activation such as suppression of neuronal ferroptosis [43], we additionally employed phenelzine, a hydrazine-based carbonyl scavenger, as a mechanistically distinct complementary approach to reduce the aldehyde burden in vivo. Direct aldehyde scavenging with hydrazine compounds represents an alternative therapeutic approach for PD [17]. Phenelzine directly scavenges aldehydes in the brain, as supported by its blood–brain barrier permeability [44] and our mass spectrometric detection of phenelzine–aldehyde conjugates in brain tissue. Beyond direct aldehyde scavenging, phenelzine has been reported to exert neuroprotective actions through inhibition of monoamine oxidase (MAO), GABA transaminase, and primary amine oxidase (PrAO) [45]. Since MAO and PrAO catalyze the formation of aldehydes such as DOPAL, 3,4-dihydroxyphenylglycolaldehyde, 5-hydroxyindoleacetaldehyde, formaldehyde, and methylglyoxal, their inhibition reduces aldehyde production and consequently lowers the levels of these aldehydes [46], [47], [48]. Importantly, strong evidence indicates that hydrazine-mediated carbonyl scavenging activity is a central mechanism underlying its neuroprotective effects. A structurally similar MAO inhibitor lacking the hydrazine group failed to replicate phenelzine's efficacy, whereas a phenelzine analogue lacking MAO activity but retaining aldehyde-scavenging capacity still afforded robust protection [49], [50]. Although other pharmacological contributions of Alda-1 and phenelzine cannot be completely excluded, these two mechanistically distinct strategies, which act on different primary targets but converge on reducing reactive aldehydes, both decreased the levels of aldehyde–protein adducts, attenuated α-synuclein aggregation, and protected against neurodegeneration in the 6-OHDA-induced rat model of PD. This convergence provides compelling evidence that aldehyde-mediated covalent protein modification contributes to dopaminergic neuron loss in this PD model, and suggests that scavenging or metabolic enhancement of aldehydes represents a viable therapeutic strategy for PD.

Neurodegeneration in PD arises from a dynamic network of multiple interacting mechanisms, including α-synuclein aggregation, oxidative stress, mitochondrial dysfunction, and neuroinflammation [51]. As non-specific electrophiles, aldehydes can covalently modify a broad range of proteins and have been implicated in multiple pathological processes relevant to dopaminergic neuronal death. For example, several aldehydes (e.g., DOPAL, 4-HNE, and acrolein) can promote α-synuclein aggregation in vitro and in vivo [14], [35], [52], [53]. α-Synuclein aggregates further induce neurotoxicity through multiple cellular processes, such as NLRP3 inflammasome-mediated neuroinflammation, actin-mediated mitophagy impairment, and ferroptosis via the STAT3/HIF-1α pathway [54], [55], [56]. Beyond promoting aggregation, α-synuclein covalently modified by aldehydes such as pyruvaldehyde has been shown to activate microglia and induce neuroinflammatory responses in vitro [57]. In addition, individual aldehydes can directly affect other key pathological events in PD through distinct pathways. Several aldehydes (e.g., DOPAL, acrolein, and acetaldehyde) can induce mitochondrial dysfunction [58], [59], [60]. Acrolein and 4-HNE can directly activate glial cells, leading to inflammatory responses [61], [62], [63]. 4-HNE can cause proteasomal dysfunction by covalent modification [64]. Consistent with these multifaceted effects of aldehydes, our study demonstrates that co-injection of multiple reactive aldehydes into the rat substantia nigra induced dopaminergic neuronal death, accompanied by α-synuclein aggregation, microglial activation, and mitochondrial dysfunction. Notably, beyond affecting individual pathways, aldehydes can modulate crosstalk among these pathogenic events. One example is neuron–glia crosstalk. On the one hand, aldehydes can activate glial cells, either directly or via covalently modified proteins, and these activated glial cells then release pro-inflammatory cytokines that further damage dopaminergic neurons. On the other hand, aldehydes directly injure dopaminergic neurons, and injured neurons can in turn activate surrounding microglia not only by releasing damage-associated molecular patterns (DAMPs), such as α-synuclein aggregates [57], [65], but also through other mechanisms such as chemokine signaling. For example, DOPAL-induced neuronal damage triggers the release of the neuronal chemokine CCL21, which activates microglia via the CCL21–CCR7 axis and exacerbates neuronal injury through neuroinflammation [66]. Another example is the vicious cycle between α-synuclein aggregation and impaired mitophagy. α-Synuclein aggregates can impair mitophagic flux, and impaired mitophagy can hinder the clearance of α-synuclein aggregates [55]. Thus, aldehyde-induced dopaminergic neuronal death in PD may not be mediated by a single, well-defined signaling pathway, but rather by the convergence of several interrelated pathogenic processes induced by multiple aldehydes. As upstream drivers of multiple pathogenic events, aldehydes play a critical role in PD etiology. However, due to the inherent complexity of endogenous aldehydes and the multifactorial nature of neuronal cell death, the relative contribution of individual aldehyde species and the causal relationships among these pathogenic processes in PD remain to be determined in future studies.

## 5 Conclusion

In this study, 40 aldehydes were identified in the SN and striatum of both 6-OHDA and rotenone-induced rat models of PD, including 22 species detected in the rat brain for the first time. Among these, 32 aldehydes were found to covalently modify proteins. In addition to lipid peroxidation, aldehydes were derived from amine oxidation and other sources. Twelve aldehydes covalently modified α-synuclein in 6-OHDA-treated rats, subsequently driving its crosslinking and aggregation. The "additive effects" of covalent modification by individual aldehydes contributed to dopaminergic neuron death. In summary, multiple biogenic aldehydes, especially those derived from abnormal amine oxidation, represent a previously underappreciated class of toxic molecules that fundamentally contribute to the progression of neurodegeneration in PD.

## CRediT authorship contribution statement

**Lin Chen:** Formal analysis, Investigation, Methodology, Visualization, Writing – original draft. **Haiping Deng:** Investigation, Methodology. **Ni Li:** Investigation, Methodology. **Ya He:** Investigation, Methodology. **Guohong Zhai:** Investigation, Methodology. **Hong Pan:** Project administration. **Chao Fang:** Conceptualization. **Fuguo Shi:** Conceptualization, Data curation, Funding acquisition, Project administration, Writing – review & editing.

## Data availability

Data will be made available on request.

## Declaration of competing interest

The authors declare that they have no known competing financial interests or personal relationships that could have appeared to influence the work reported in this paper.
