# Supplementary material for: Protein adduction by multiple aldehydes contributes to α-synuclein aggregation and dopaminergic neuron loss in rat models of Parkinson's disease
Source: Redox Biol. 2026 Nov 2;98:104468. doi: 10.1016/j.redox.2026.104468 (PMC13756803; doi:10.1016/j.redox.2026.104468)
Supplement: Supplementary file 1 [file mmc1.docx]

**Supplementary materials:**

**Protein adduction by multiple aldehydes contributes to α-synuclein aggregation and dopaminergic neuron loss in rat models of Parkinson's disease**

Lin Chen^a^, Haiping Deng^a^, Ni Li^a^, Ya He^a^, Guohong Zhai^a^, Hong Pan^a^, Chao Fang^b^, Fuguo Shi^a^*

*a* *Key Laboratory of Basic Pharmacology of Ministry of Education & Joint International Research Laboratory of Ethnomedicine of Ministry of Education, Zunyi Medical University, Zunyi 563003, China*

*b Department of Pharmacy, Tongren Hospital, Shanghai Jiao Tong University School of Medicine, Shanghai 200025, China*

**Correspondence: Fuguo Shi ([fuguoshi@hotmail.com](mailto:fuguoshi@hotmail.com))*, *Key Laboratory of Basic Pharmacology of Ministry of Education & Joint International Research Laboratory of Ethnomedicine of Ministry of Education, Zunyi Medical University, Xinpu District, Zunyi 563003, China. Tel: 86-0851-28642303 Fax: 86-0851-28642303.*

**Table of contents**

***Supplementary methods:***

**1 Chemicals and reagents**

**2 Parallel homogenization control experiment**

**3 Determination of ATP levels**

***Supplementary results:***

**1** Identification of free aldehydes by isotope labeling and LC-MS method

**2** Structural elucidation of representative conjugates of aldehydes with free amino acids

**3** Structural elucidation of representative conjugates of aldehydes with protein amino acid residues

**4** Structural elucidation of representative conjugates of aldehydes with phenelzine

**5** Structural elucidation of representative conjugates of aldehydes with α-synuclein peptide fragments

***Supplementary data:***

**Figures**

**Fig. S1** Extracted ion chromatograms, MS spectra, and MS/MS spectra of representative BEPC-labeled aldehydes found in the brain of 6‑OHDA‑treated rats.

**Fig. S2** Chemical structures of aldehydes found in the brain of 6‑OHDA‑treated rats.

**Fig. S3** Extracted ion chromatograms and MS/MS spectra of representative BEPC‑labeled aldehydes from the brain of 6‑OHDA‑treated rats and of the corresponding BEPC‑labeled authentic standards.

**Fig. S4** Extracted ion chromatograms and MS/MS spectra of representative HTP‑labeled aldehydes from the brain of 6‑OHDA‑treated rats and of the corresponding HTP‑labeled authentic standards.

**Fig. S5** Extracted ion chromatograms and MS/MS spectra of representative conjugates of aldehydes with free amino acids in the brain of 6‑OHDA‑treated rats.

**Fig. S6** Extracted ion chromatograms and MS/MS spectra of representative aldehyde‑protein amino acid residue conjugates in the brain of 6‑OHDA‑treated rats (upper panel) and the corresponding conjugates from in vitro incubation (lower panel).

**Fig. S7** Extracted ion chromatograms and MS/MS spectra of representative conjugates of 4‑HNE with different amino acid residues in the brain of 6‑OHDA‑treated rats.

**Fig. S8** Comparison of 0.9% NaCl versus DFO/BHT‑supplemented 0.9% NaCl for homogenization on aldehyde–protein adduct levels in the substantia nigra at 4 and 6 h after 6‑OHDA injection.

**Fig. S9** MS/MS spectra of representative phenelzine‑aldehyde conjugates in the brain of 6‑OHDA‑treated rats.

**Fig. S10** Behavioral examinations in 6‑OHDA‑treated rats after modulating aldehyde levels by Alda-1 or phenelzine treatment.

**Fig. S11** Microglial cell activation in rats receiving direct injection of aldehydes.

**Fig. S12** Mitochondrial function in rats receiving direct injection of aldehydes.

**Fig. S13** Extracted ion chromatograms and MS/MS spectra of representative conjugates of 4‑HNE with two AK peptides of α‑synuclein in the brains of 6‑OHDA‑treated rats.

**Tables**

**Tab. S1** Aldehydes identified in the brain of 6‑OHDA‑treated rats.

**Tab. S2** Representative conjugates of aldehydes with free amino acids in the brain of 6‑OHDA‑treated rats.

**Tab. S3** Representative conjugates of aldehydes with protein amino acid residues in the brain of 6‑OHDA‑treated rats.

**Tab. S4** Changes in the levels of aldehyde‑protein amino acid residue conjugates in the substantia nigra between the 6‑OHDA group and the sham group at 6 h post-injection.

**Tab. S5** Changes in the levels of aldehyde‑protein amino acid residue conjugates in the substantia nigra between the rotenone group and the sham group at 3 d post-injection.

**Tab. S6** Changes in the levels of aldehyde‑protein amino acid residue conjugates in the substantia nigra between the rotenone group and the sham group at 7 d post-injection.

**Tab. S7** Changes in the levels of aldehyde‑protein amino acid residue conjugates in the substantia nigra between the 6‑OHDA+Alda‑1 group and the 6‑OHDA group at 6 h post-injection.

**Tab. S8** Changes in the levels of aldehyde‑protein amino acid residue conjugates in the substantia nigra between the 6‑OHDA+phenelzine group and the 6‑OHDA group at 6 h post-injection.

**Tab. S9** Aldehyde‑modified α‑synuclein peptide fragments in the brains of 6‑OHDA‑treated rats.

**Supplementary methods:**

**1. Chemicals and reagents**

6-Hydroxydopamine hydrochloride, rotenone, calcium chloride, DL-Dithiothreitol, acetic acid, iodoacetamide, L-ascorbic acid, and protease (type XⅣ bacterial from *strepto*) were obtained from Sigma-Aldrich (St Louis, MO, USA). 3,4-Dihydroxyphenylacetaldehyde (DOPAL) was purchased from Toronto Research Chemicals Inc. (Toronto, Canada). 4-Bromo-1-ethyl-1H-pyrazole-3-carbohydrazide (BEPC), 2-Hydrazino-4-(trifluoromethyl)-pyrimidine (HTP), cuminaldehyde, pentanal, 4-hydroxybenzaldehyde, and butylated hydroxytoluene (BHT) were purchased from J&K Chemicals (Beijing, China). Trypsin (Mass Spectrometry Grade) was purchased from Beijing Shengxia Proteins Scientific Ltd (Beijing, China). Recombinant rat α-synuclein protein (NBP3-14774) was obtained from Novus Biologicals (Centennial, CO, USA). Acetaldehyde, crotonaldehyde, glyoxal, 4-hydroxynonenal (4-HNE), lactaldehyde, 2-phenylpropanal, pyridoxal, and desferrioxamine (DFO) were obtained from Shanghai Macklin Biochemical Co., Ltd. (Shanghai, China). Malonic semialdehyde, 2-Decenal, homovanillin, 4-hydroxyhexenal (4-HHE), malondialdehyde (MDA), methylimidazole acetaldehyde, 2-nonenal, pyruvaldehyde, butanal, formaldehyde, succinaldehyde, acrolein, Alda-1, benzaldehyde, 4-hydroxyphenylacetaldehyde, 5-hydroxyindoleacetaldehyde, nonanal, octanal, 2-octenal, phenelzine sulfate, and propanal were obtained from Shanghai Aladdin Biochemical Technology Co., Ltd. (Shanghai, China). Methanol and acetonitrile (LC-MS grade) were purchased from Thermo Fisher Scientific (Fair Lawn, NJ, USA).

**2. Parallel homogenization control experiment**

A parallel homogenization comparison experiment was performed to exclude potential ex vivo autoxidation artifacts. The substantia nigra tissues from two independent groups of rats were used for the two homogenization protocols, respectively. The first protocol followed the routine homogenization in ice‑cold 0.9% NaCl without protective additives, and the second employed a protective buffer (ice‑cold 0.9% NaCl containing 1 mM DFO and 200 µM BHT). For both protocols, substantia nigra tissues were collected at 0, 4, and 6 h after 6‑OHDA injection (n = 6 per time point per protocol). The homogenate was immediately precipitated with three volumes of ice‑cold methanol, and the resulting protein pellet was washed twice with ice‑cold methanol to remove residual lipids before being subjected to the identical enzymatic digestion and UPLC‑Q/Orbitrap MS analysis described in the main Methods section.

**3.** **Determination of ATP levels**

ATP levels in the striatum were measured using commercial kits from Solarbio Science & Technology Co., Ltd. (BC0515 and BC0300, Beijing, China). The assays were performed according to the manufacturers' protocol.

**Supplementary results:**

**1. Identification of free aldehydes by isotope labeling and LC-MS method**

In this study, 4-Bromo-1-ethyl-1H-pyrazole-3-carbohydrazide (BEPC) labeling combined with LC-MS method was used for recognition of free aldehydes in the brain. The extracted ion chromatograms, MS spectra, and MS/MS spectra of BEPC-labeled 4-hydroxybenzaldehyde, benzaldehyde, and glyoxal were exhibited in Fig. S1. Unique ^79^Br/^81^Br isotope feature can be observed in the extracted ion chromatograms, MS spectra, and MS/MS spectra of BEPC-labeled aldehydes. BEPC-labeled aldehydes can be easily distinguished by the unique isotope feature.

BEPC-labeled benzaldehyde was an example to illustrate the aldehyde recognition (Fig. S3). A pair of peaks with identical retention time (13.86 min) and peak shape and a 1:1 peak area ratio was observed in the extracted ion chromatogram of *m/z* 321.0346 and 323.0326. Two isotopic pairs of fragment ions at *m/z* 200.9656/202.9636 and 218.9761/220.9741 (base peak) were found in the MS/MS spectra of the precursor ions at *m/z* 321.0346 and 323.0326, resulting from the cleavage of the fragile amide bond. Product ions at *m/z* 218.9761 and 220.9741 were water adducts of the fragment ions at *m/z* 200.9656 and 202.9636, respectively. The cleavage of the N-N bond generated the diagnostic ion at *m/z* 104.0496 in the aldehydes portion. BEPC-labeled benzaldehyde can be recognized and identified by the unique Br isotopic feature and diagnostic fragment ions. By this method, 40 aldehydes were recognized and identified in the substantia nigra and striatum of 6-OHDA-treated rats. The chemical structures of 30 aldehydes were finally confirmed by standard compounds. As shown in Fig. S3, the BEPC-labeled aldehydes obtained from incubation of aldehyde standards with BEPC exert the same chromatographic behavior and fragmentation pattern as conjugates found in rat brain samples.

HTP derivatization was further used for verification of aldehyde structure identification. Here, HTP-labeled nonanal was an example to illustrate the verification (Fig. S4). In the EIC of *m/z* 303.1791, a peak with retention time at 32.93 min was observed. The mass-to-charge ratio was 124.1252 Da higher than that of HTP (*m/z* 178.0466), suggesting it may be nonanal. In the MS/MS spectrum of *m/z* 303.1791, the characteristic ion at *m/z* 164.0424 was derived from the HTP portion and the diagnostic ion at *m/z* 152.0700 was derived from the aldehyde portion after the cleavage of the N-N bond. The HTP-nonanal derivative obtained from the incubation of nonanal standard with HTP exerts the same chromatographic behavior and fragmentation pattern as those of the derivative found in rat brain samples, which provided strong evidence for structure elucidation.

**2 Structural elucidation of representative conjugates of aldehyde with free amino acids**

Pyruvaldehyde-glutamic acid conjugate and acrolein-lysine conjugate were used to demonstrate the conjugates of aldehydes with free amino acids (Fig. S5). In the EIC of *m*/*z* 202.0710, a peak with retention time at 2.54 min was observed. The mass-to-charge ratio of the precursor ion was 129.0426 Da higher than that of pyruvaldehyde (*m*/*z* 72.0211). In its MS/MS spectrum, the product ions at *m/z* 156.0658, 138.0553, and 110.0606 were derived from the successive loss of HCOOH, HCOOH+H_2_O, and 2HCOOH, respectively. Such fragmentation behavior was due to two HCOOH moieties in the glutamic acid. For the acrolein-lysine conjugate, the product ions at *m/z* 145.0978, 130.0852, 116.0706, and 70.0657 were characteristic fragments of lysine. The fragment ions at *m/z* 126.1267 resulted from the loss of HCOOH+NH_2_ moieties. In summary, the conjugates of aldehydes with free amino acids were identified by high-resolution molecular mass and the diagnostic product ions in the MS/MS spectra.

**3 Structural elucidation of representative conjugates of aldehyde with protein amino acid residues**

Protein adductions by aldehydes were characterized by the conjugates of aldehydes with the single amino acid residue of proteins after exhaustive proteolytic digestion. In general, three types of diagnostic product ions supported the structure elucidation. The first type was the shared fragments from the amino acid portion, such as the ions at *m/z* 130.0868, 116.0712, and 84.0808 derived from the fragmentation of a lysine residue. The second type of fragment was the ions resulting from the cleavage of the fragile C-N bond. The third type of product ions was from the aldehyde portion. The conjugates of amino acid residue with four representative aldehydes were exhibited in Fig. S6. In the proteolytic rat brain samples, a peak with retention time at 10.92 min was detected in the EIC of *m/z* 253.1659. This precursor ion was 128.0949 Da (molecular weight of lysine-H_2_O, 146.1055-18.0106) higher than that of methylimidazole acetaldehyde (*m/z* 124.0637), suggesting the formation of methylimidazole acetaldehyde-lysine residue conjugate. In the MS/MS spectrum, the product ions at *m/z* 130.0868, 116.0721, and 84.0815 were characteristic fragments of lysine. The fragment ions at *m/z* 192.1492 resulted from the loss of HCOOH+NH_2_ moieties. The daughter ion at *m/z* 97.0758 resulted from methylimidazole acetaldehyde portion after cleavage of the C-C bond. This high-resolution precursor ion and characteristic fragments indicated the formation of methylimidazole acetaldehyde-lysine residue conjugate.

A peak with a retention time of 7.39 min was observed in the EIC of *m/z* 251.1391. This precursor ion was 128.0949 Da (molecule weight of lysine-H_2_O, 146.1055-18.0106) higher than 4-hydroxybenzaldehyde (*m/z* 122.0368). In the MS/MS spectrum, the fragment ion at *m/z* 120.0440 was derived from the aldehyde portion. The ions at *m/z* 130.0865 and 84.0813 were derived from the lysine portion. The fragment ions at *m/z* 192.1389 resulted from the loss of HCOOH+NH_2_ groups. These ions supported the formation of 4-hydroxybenzaldehyde-lysine residue conjugate. A peak with retention time at 3.85 min in the EIC of *m/z* 199.1077 was assigned as crotonaldehyde-glutamine residue conjugate. In the MS/MS spectrum, the diagnostic ion at *m/z* 70.0659 was derived from the aldehyde portion after the cleavage of the C-N bond. The ion at *m/z* 84.0450 was a characteristic fragment of glutamine. The fragment at *m/z* 153.1026 was generated from the loss of HCOOH. In the EIC of *m/z* 221.0921, a peak with retention time at 5.03 min was found and thought to be benzaldehyde-asparagine residue conjugate. In the MS/MS spectrum, the fragment ion at *m/z* 132.0447 was derived from the aldehyde portion, and the fragment ion at *m/z* 88.0401 was derived from the asparagine portion. The fragment ions at *m/z* 175.0871 and 158.0605 resulted from the loss of HCOOH and HCOOH+NH_2_, respectively. Three types of characteristic product ions generated from conjugates of aldehydes and amino acid residues provided strong evidence for structure elucidation. In the present study, 32 aldehydes were found that can covalently modify proteins in the brains of 6-OHDA-treated rats (Tab. S3).

The conjugates derived from the covalent binding of 4-HNE with different amino acid residues were exhibited in Fig. S7. They were 4-HNE-modified lysine residue, 4-HNE-modified asparagine residue, and glutamine-4-HNE-lysine crosslink. In the proteolytic rat brain samples, the peak of *m/z* 267.2067 and retention time at 6.75 min was assigned to be 4-HNE-lysine pyrrole conjugate. In the MS/MS spectrum, the fragment at *m/z* 138.1277 was an important diagnostic ion derived from the pyrrole portion after the cleavage of the C-N bond. The product ions at *m/z* 116.0706 and 84.0812 were characteristic fragments of lysine. The ions at *m/z* 223.2166 and 206.1902 were generated from the loss of HCOOH and HCOOH+NH_2_, respectively. These characteristic fragments provided strong evidence for the formation of 4-HNE-lysine pyrrole conjugate. 4-HNE-asparagine pyrrole conjugate (*m/z* 253.1547) was found at a retention time of 8.39 min. In the MS/MS spectrum, the fragment at *m/z* 138.1282 was an important diagnostic ion derived from the pyrrole portion after the cleavage of the C-N bond. The fragment ion at *m/z* 207.1489 was generated from the loss of HCOOH. 4-HNE contains two electrophilic reaction sites (aldehyde group and α, β-unsaturated bond), therefore, 4-HNE can simultaneously react with two amino acid residues. A 4-HNE-derived lysine-glutamine crosslinking (retention time=17.47 min) was found in the EIC of *m/z* 431.2864. The fragment at *m/z* 300.1923 was an important ion derived from the aldehyde portion after the cleavage of the C-N bond. The fragment ion at *m/z* 171.1496 was an important diagnostic ion derived from the 4-HNE portion after the cleavage of the amide bond and the C-N bond. The product ions at *m/z* 132.1022 and 116.0711 were characteristic fragments of lysine.

**4 Structural elucidation of representative conjugates of aldehyde with phenelzine**

Phenelzine contains a hydrazine group and enables nucleophilic trapping of reactive aldehydes. In this study, the conjugates of phenelzine with various aldehydes were detected in the brain samples after administration of phenelzine. The MS/MS spectra of representative phenelzine-aldehyde conjugates are shown in Fig. S8. For the phenelzine-pyruvaldehyde conjugate, its precursor ion was at *m/z* 191.1170. The fragment ions at *m/z* 122.0960 and *m/z* 70.0288 were derived from the phenelzine and pyruvaldehyde portion after the cleavage of the N-N bond, respectively. The fragment at *m/z* 103.0536 was also a feature fragment of phenelzine. In the MS/MS spectrum of the phenelzine-glyoxal conjugate, the fragment ion at *m/z* 122.0958 was derived from the phenelzine portion after the cleavage of the N-N bond. The ion at *m/z* 73.0388 was an important diagnostic ion derived from the glyoxal portion after the cleavage of the C-N bond. The fragment ion at *m/z* 103.0536 was the shared ion of the phenelzine portion. In the MS/MS spectrum of the conjugate of phenelzine with pyridoxal 5’-phosphate, the fragment ion at *m/z* 122.0954 was derived from the phenelzine portion. The ion *m/z* 245.0317 was derived from the pyridoxal 5’-phosphate portion after the cleavage of the N-N bond. The fragment at *m/z* 165.0548 was derived from the loss of phosphate moiety in pyridoxal 5’-phosphate.

**5 Structural elucidation of representative conjugates of aldehydes with α-synuclein peptide fragments**

**5.1 The pyruvaldehyde-modified GLSK peptide fragment**

The peak with retention time at 17.00 min was found in the extracted ion chromatogram of *m*/*z* 458.2609 in extracted rat brain AS samples, whose molecular mass was 54.0105 Da higher than that of GLSK peptide (*m*/*z* 404.2504). In the MS/MS spectra of the pyruvaldehyde-GLSK conjugate, the +54.01 Da mass shift was observed in the y-type fragments, indicating the covalent binding of the lysine residue of GLSK with pyruvaldehyde through the Schiff base reaction. The cleavage of the (γ)C-(δ)C bond in the lysine portion to lose pyruvaldehyde+C_2_H_7_N was a major fragmentation pathway of the conjugate, resulting in several major product ions such as 341.1813 and 187.1076. The structure of GLSK-pyruvaldehyde conjugate was verified by the *in vitro* incubation of pyruvaldehyde with recombinant rat AS.

**5.2** **The AK-4-HNE-AK crosslink**

A 4-HNE-derived crosslink of AK-AK of *m/z* 573.3970 was observed in the brains of 6-OHDA-treated rat, and the retention time was 16.10 min (Fig. S10). In the MS/MS spectrum, the fragment ion at *m/z* 399.2968 was y1* ion minus C_3_H_8_N+COOH after cleavage of the γC-δC bond. Product ion at *m/z* 373.2807 resulted from the loss of AK of precursor ion, and *m/z* 314.2075 resulted from the loss of C_3_H_9_N of *m/z* 373.2807. The fragment ion at *m/z* 260.1971 and 201.1235 were peptide fragment AK plus C_3_H_6_ and minus ε-NH_3_ in lysine, respectively. The product ion at *m/z* 147.1130 was the molecular weight of peptide fragment K (lysine), while *m/z* 173.1285 was based on peptide fragment K plus C_2_H_4_, *m/z* 129.1024 was K-H_2_O.

**Supplementary data:**

**Fig. S1** Extracted ion chromatograms, MS spectra, and MS/MS spectra of representative BEPC-labeled aldehydes found in the brain of 6‑OHDA‑treated rats.

**Fig. S2** Chemical structures of aldehydes found in the brain of 6‑OHDA‑treated rats. Note: The aldehyde marked with symbol **#** or & indicated that it can bind with either free amino acids or proteins. The aldehyde marked with asterisk* indicated it can bind to both free amino acids and proteins. The aldehyde marked with symbol§ indicated it was verified by authentic standard.

**Fig. S3** Extracted ion chromatograms and MS/MS spectra of representative BEPC‑labeled aldehydes from the brain of 6‑OHDA‑treated rats and of the corresponding BEPC‑labeled authentic standards.

**Fig. S4** Extracted ion chromatograms and MS/MS spectra of representative HTP‑labeled aldehydes from the brain of 6‑OHDA‑treated rats and of the corresponding HTP‑labeled authentic standards.

**Fig. S5** Extracted ion chromatograms and MS/MS spectra of representative conjugates of aldehydes with free amino acids in the brain of 6‑OHDA‑treated rats.

**Fig. S6** Extracted ion chromatograms and MS/MS spectra of representative aldehyde‑protein amino acid residue conjugates in the brain of 6‑OHDA‑treated rats (upper panel) and the corresponding conjugates from *in vitro* incubation(lower panel).

**Fig. S7** Extracted ion chromatograms and MS/MS spectra of representative conjugates of 4‑HNE with different amino acid residues in the brain of 6‑OHDA‑treated rats.

**Fig. S8** Comparison of 0.9% NaCl versus DFO/BHT‑supplemented 0.9% NaCl for homogenization on aldehyde–protein adduct levels in the substantia nigra at 4 and 6 h after 6‑OHDA injection (n = 6 per time point for each protocol). n.s., not significant.

**Fig. S9** MS/MS spectra of representative phenelzine-aldehyde conjugates in the brain of 6‑OHDA‑treated rats.

**Fig. S10** Behavioral examinations in 6‑OHDA‑treated rats after modulating aldehyde levels by Alda-1 or phenelzine treatment. (A) Open-field; (B) Rotarod tests. Rats were injected with 6-OHDA, and then received intraperitoneal injection of 50 mg/kg Alda-1 (first dose 1 h before 6-OHDA) or 15 mg/kg phenelzine (first dose 1 h after 6-OHDA) once daily for 3 consecutive days. Behavioral examinations were performed at 14 days after 6-OHDA injection (n = 10). ***P*<0.01.

**Fig. S11** Microglial cell activation in rats receiving direct injection of aldehydes. (A) Representative immunohistochemical staining and (B) quantification of Iba-1-positive microglia in the substantia nigra (n = 6). Scale bar = 100 μm. The aldehyde mixture (acrolein, 4‑HNE, DOPAL, MDA, pyruvaldehyde, crotonaldehyde, and malonic semialdehyde, each at 0.5 nmol) was injected into the left substantia nigra of rats. The substantia nigra was collected at 72 h after injection of aldehydes. The data are presented as the mean ± SD. ***P*<0.01.

**Fig. S12** Mitochondrial function in rats receiving direct injection of aldehydes. (A) ATP levels in the striatum measured by ELISA (n = 6). (B) PINK1 and (C) SQSTM1 protein levels in the substantia nigra measured by western blotting (n = 6). The aldehyde mixture (acrolein, 4‑HNE, DOPAL, MDA, pyruvaldehyde, crotonaldehyde, and malonic semialdehyde, each at 0.5 nmol) was injected into the left substantia nigra of rats. The substantia nigra and striatum were collected at 72 h after injection of aldehydes. The data are presented as the mean ± SD. ***P*<0.01.

**Fig. S13** Extracted ion chromatograms and MS/MS spectra of representative conjugate of 4‑HNE with two AK peptides of α‑synuclein in the brains of 6‑OHDA‑treated rats.

**Tab. S1** Aldehydes identified in the brain of 6‑OHDA‑treated rats.

| No. | Tested mass (*m/z*) | Calculated mass (*m/z*) | Formulas | Compound name | HMDB ID | CAS | Source |
| --- | --- | --- | --- | --- | --- | --- | --- |
| 1 | 30.0102 | 30.0106 | CH_2_O | Formaldehyde* | HMDB0001426 | 50-00-0 | Lipid peroxidation |
| 2 | 44.0255 | 44.0262 | C_2_H_4_O | Acetaldehyde* | HMDB0000990 | 75-07-0 | Lipid peroxidation |
| 3 | 56.0259 | 56.0262 | C_3_H_4_O | Acrolein* | HMDB0041822 | 107-02-8 | Lipid peroxidation |
| 4 | 58.0053 | 58.0055 | C_2_H_2_O_2_ | Glyoxal* | HMDB0303107 | 107-22-2 | Glycosylation |
| 5 | 58.0414 | 58.0419 | C_3_H_6_O | Propanal* | HMDB0003366 | 123-38-6 | Lipid peroxidation |
| 6 | 70.0415 | 70.0419 | C_4_H_6_O | Crotonaldehyde* | HMDB0034233 | 123-73-9 | Lipid peroxidation |
| 7 | 72.0205 | 72.0211 | C_3_H_4_O_2_ | MDA* | HMDB0006112 | 542-78-9 | Lipid peroxidation |
| 8 | 72.0208 | 72.0211 | C_3_H_4_O_2_ | Pyruvaldehyde* | HMDB0001167 | 78-98-8 | Glycosylation |
| 9 | 72.0573 | 72.0575 | C_4_H_8_O | Butanal* | HMDB0003543 | 123-72-8 | Lipid peroxidation |
| 10 | 74.0361 | 74.0368 | C_3_H_6_O_2_ | Lactaldehyde*^, #^ | HMDB0003052 | 598-35-6 | Glycosylation |
| 11 | 84.0210 | 84.0211 | C_4_H_4_O_2_ | 2-Butenedial^#^ | HMDB0247804 | 3675-13-6 | Unknown |
| 12 | 86.0365 | 86.0368 | C_4_H_6_O_2_ | Succinaldehyde*^, #^ | HMDB0258537 | 638-37-9 | Unknown |
| 13 | 86.0730 | 86.0732 | C_5_H_10_O | Pentanal* | HMDB0031206 | 110-62-3 | Lipid peroxidation |
| 14 | 88.0156 | 88.0160 | C_3_H_4_O_3_ | Malonic semialdehyde*^, #^ | HMDB0011111 | 926-61-4 | Amine metabolism |
| 15 | 96.0208 | 96.0211 | C_5_H_4_O_2_ | 2-Furancarboxaldehyde^#^ | HMDB0032914 | 98-01-1 | Unknown |
| 16 | 98.0366 | 98.0368 | C_5_H_6_O_2_ | 2-Pentenedial^#^ | HMDB0252802 | 821-42-1 | Unknown |
| 17 | 100.0516 | 100.0524 | C_5_H_8_O_2_ | Glutaraldehyde^#^ | HMDB0029599 | 111-30-8 | Unknown |
| 18 | 106.0416 | 106.0419 | C_7_H_6_O | Benzaldehyde*^, #^ | HMDB0006115 | 100-52-7 | Amine metabolism |
| 19 | 110.0365 | 110.0368 | C_6_H_6_O_2_ | 5-Methyl-2-furancarboxaldehyde^#^ | HMDB0033002 | 620-02-0 | Unknown |
| 20 | 114.0674 | 114.0681 | C_6_H_10_O_2_ | 5-Oxohexanal^#^ | HMDB0033568 | 505-03-3 | Unknown |
| 21 | 114.0679 | 114.0681 | C_6_H_10_O_2_ | 4-HHE* | -- | 17427-21-3 | Lipid peroxidation |
| 22 | 122.0365 | 122.0368 | C_7_H_6_O_2_ | 4-Hydroxybenzaldehyde*^, #^ | HMDB0011718 | 123-08-0 | Amine metabolism |
| 23 | 124.0632 | 124.0637 | C_6_H_8_N_2_O | Methylimidazole acetaldehyde*^, #^ | HMDB0004181 | 19639-03-3 | Amine metabolism |
| 24 | 126.1042 | 126.1045 | C_8_H_14_O | 2-Octenal*^, #^ | HMDB0030961 | 2363-89-5 | Lipid peroxidation |
| 25 | 128.1201 | 128.1201 | C_8_H_16_O | Octanal* | HMDB0001140 | 124-13-0 | Lipid peroxidation |
| 26 | 134.0368 | 134.0368 | C_8_H_6_O_2_ | Phenylglyoxal^#^ | HMDB0061916 | 1074-12-0 | Unknown |
| 27 | 134.0729 | 134.0732 | C_9_H_10_O | 2-Phenylpropanal*^, #^ | HMDB0031626 | 93-53-8 | Amine metabolism |
| 28 | 136.0522 | 136.0524 | C_8_H_8_O_2_ | 4-Hydroxyphenylacetaldehyde*^, #^ | HMDB0003767 | 7339-87-9 | Amine metabolism |
| 29 | 140.1196 | 140.1201 | C_9_H_16_O | 2-Nonenal*^, #^ | HMDB0031269 | 2463-53-8 | Lipid peroxidation |
| 30 | 142.1358 | 142.1358 | C_9_H_18_O | Nonanal* | HMDB0059835 | 124-19-6 | Lipid peroxidation |
| 31 | 148.0887 | 148.0888 | C_10_H_12_O | Cuminaldehyde*^, #^ | HMDB0002214 | 122-03-2 | Unknown |
| 32 | 152.0470 | 152.0473 | C_8_H_8_O_3_ | DOPAL* | HMDB0003791 | 5707-55-1 | Amine metabolism |
| 33 | 154.1352 | 154.1358 | C_10_H_18_O | 2-Decenal*^, #^ | HMDB0030999 | 3913-71-1 | Lipid peroxidation |
| 34 | 156.1153 | 156.1150 | C_9_H_16_O_2_ | 4-HNE* | HMDB0004362 | 75899-68-2 | Lipid peroxidation |
| 35 | 166.0631 | 166.0630 | C_9_H_10_O_3_ | Homovanillin*^, #^ | HMDB0005175 | 5703-24-2 | Amine metabolism |
| 36 | 167.0574 | 167.0582 | C_8_H_9_NO_3_ | Pyridoxal* | HMDB0001545 | 66-72-8 | Vitamin metabolism |
| 37 | 175.0633 | 175.0638 | C_10_H_9_NO_2_ | 5-Hydroxyindoleacetaldehyde* | HMDB0004073 | 1892-21-3 | Amine metabolism |
| 38 | 194.0435 | 194.0427 | C_6_H_10_O_7_ | Galacturonic acid^#^ | HMDB0002545 | 14982-50-4 | Unknown |
| 39 | 208.0593 | 208.0583 | C_7_H_12_O_7_ | Methyl galacturonate^#^ | HMDB0302222 | 4120-73-4 | Unknown |
| 40 | 247.0238 | 247.0246 | C_8_H_10_NO_6_P | Pyridoxal 5'-phosphate | HMDB0001491 | 54-47-7 | Vitamin metabolism |

* Indicates the compound has been verified by authentic standard.

^#^ Indicates the compound is found in rat brain for the first time.

**Tab. S2** Representative conjugates of aldehydes with free amino acids in the brain of 6‑OHDA‑treated rats.

| Conjugates | Formula | RT (min) | Calculated mass (*m/z*) | Tested mass (*m/z*) | Error (ppm) |
| --- | --- | --- | --- | --- | --- |
| Acrolein-Lys | C_9_H_16_N_2_O_2_ | 8.27 | 184.1212 | 184.1209 | 1.63 |
| Crotonaldehyde-Gln | C_9_H_14_N_2_O_3_ | 2.90 | 198.1004 | 198.1002 | 1.01 |
| MDA-Glu | C_8_H_11_NO_5_ | 2.51 | 201.0637 | 201.0635 | 0.99 |
| Lactaldehyde-Gln | C_8_H_14_N_2_O_4_ | 2.21 | 202.0954 | 202.0948 | 2.97 |
| Glyoxal-Phe | C_11_H_11_NO_3_ | 13.38 | 205.0739 | 205.0735 | 1.95 |
| Acetaldehyde-Tyr | C_11_H_13_NO_3_ | 15.88 | 207.0895 | 207.0892 | 1.45 |
| 2-Butenedial-Lys | C_10_H_16_N_2_O_3_ | 2.16 | 212.1161 | 212.1157 | 1.89 |
| Malonic semialdehyde-Lys | C_9_H_16_N_2_O_4_ | 2.90 | 216.1110 | 216.1108 | 0.93 |
| Benzaldehyde-Asp | C_11_H_11_NO_4_ | 9.24 | 221.0688 | 221.0689 | -0.45 |
| 2-Pentenedial-Gln | C_10_H_14_N_2_O_4_ | 5.87 | 226.0954 | 226.095 | 1.77 |
| Glutaraldehyde-Gln | C_10_H_16_N_2_O_4_ | 8.96 | 228.1110 | 228.1109 | 0.44 |
| Butanal-Arg | C_10_H_20_N_4_O_2_ | 13.95 | 228.1586 | 228.1588 | -0.88 |
| Succinaldehyde-Phe | C_13_H_15_NO_3_ | 24.00 | 233.1052 | 233.1062 | -4.29 |
| 5-Oxohexanal-Lys | C_12_H_22_N_2_O_3_ | 5.17 | 242.1630 | 242.1625 | 2.06 |
| Pentanal-Arg | C_11_H_22_N_4_O_2_ | 10.09 | 242.1743 | 242.1751 | -3.30 |
| 4-Hydroxybenzaldehyde-Lys | C_13_H_18_N_2_O_3_ | 10.74 | 250.1317 | 250.1309 | 3.20 |
| 2-Nonenal-Asp | C_13_H_21_NO_4_ | 2.52 | 255.1471 | 255.1469 | 0.78 |
| Octanal-Lys | C_14_H_28_N_2_O_2_ | 26.81 | 256.2151 | 256.2143 | 3.12 |
| 2-Furancarboxaldehyde-Tyr | C_14_H_13_NO_4_ | 23.47 | 259.0845 | 259.0842 | 1.16 |
| Phenylglyoxal-Gln | C_13_H_14_N_2_O_4_ | 12.84 | 262.0954 | 262.0946 | 3.05 |
| 2-Phenylpropanal-Gln | C_14_H_18_N_2_O_3_ | 10.03 | 262.1317 | 262.1306 | 4.20 |
| 4-Hydroxyphenylacetaldehyde-Lys | C_14_H_20_N_2_O_3_ | 11.49 | 264.1474 | 264.1467 | 2.65 |
| Nonanal-Lys | C_15_H_30_N_2_O_2_ | 29.11 | 270.2307 | 270.2299 | 2.96 |
| Cuminaldehyde-Lys | C_16_H_24_N_2_O_2_ | 23.67 | 276.1838 | 276.1826 | 4.34 |
| DOPAL-Lys | C_14_H_20_N_2_O_4_ | 6.17 | 280.1423 | 280.1417 | 2.14 |
| Homovanillin-Lys | C_15_H_22_N_2_O_4_ | 11.88 | 294.1580 | 294.157 | 3.40 |
| Pyridoxal-Glu | C_13_H_16_N_2_O_6_ | 2.46 | 296.1008 | 296.1003 | 1.69 |
| 5-Methyl-2-furancarboxaldehyde-Trp | C_17_H_16_N_2_O_3_ | 14.15 | 296.1161 | 296.1168 | -2.36 |
| 2-Decenal-Lys | C_16_H_32_N_2_O_3_ | 31.70 | 300.2413 | 300.2401 | 4.00 |
| Methylimidazole acetaldehyde-Trp | C_17_H_18_N_4_O_2_ | 44.18 | 310.1430 | 310.1434 | -1.29 |
| Galacturonic acid-Gln | C_11_H_18_N_2_O_9_ | 2.33 | 322.1012 | 322.1024 | -3.73 |
| Methyl galacturonate-Glu | C_12_H_19_NO_10_ | 32.10 | 337.1009 | 337.0997 | 3.56 |
| Pyridoxal 5'-phosphate-Trp | C_19_H_20_N_3_O_7_P | 13.28 | 433.1039 | 433.1034 | 1.15 |

**Tab. S3** Representative conjugates of aldehydes with protein amino acid residues in the brain of 6‑OHDA‑treated rats.

| Conjugates | Formula | RT (min) | Calculated mass (*m/z*) | Tested mass (*m/z*) | Error (ppm) |
| --- | --- | --- | --- | --- | --- |
| Formaldehyde-Lys | C_7_H_14_N_2_O_2_ | 1.92 | 158.1055 | 158.1056 | -0.63 |
| Propanal-Asn | C_7_H_12_N_2_O_3_ | 7.59 | 172.0848 | 172.085 | -1.16 |
| Acrolein-Lys | C_9_H_16_N_2_O_2_ | 8.30 | 184.1212 | 184.1213 | -0.54 |
| Pyruvaldehyde-Asn | C_7_H_10_N_2_O_4_ | 11.75 | 186.0641 | 186.0640 | 0.54 |
| Glyoxal-Lys | C_8_H_14_N_2_O_3_ | 8.09 | 186.1004 | 186.1012 | -4.30 |
| Lactaldehyde-Asn | C_7_H_12_N_2_O_4_ | 8.90 | 188.0797 | 188.0796 | 0.53 |
| Crotonaldehyde-Lys | C_10_H_18_N_2_O_2_ | 3.75 | 198.1368 | 198.1367 | 0.50 |
| MDA-Lys | C_9_H_16_N_2_O_3_ | 11.37 | 200.1161 | 200.1162 | -0.50 |
| Malonic semialdehyde-Asn | C_7_H_10_N_2_O_5_ | 16.04 | 202.0590 | 202.0588 | 0.99 |
| 2-Butenedial-Gln | C_9_H_12_N_2_O_4_ | 15.58 | 212.0797 | 212.0795 | 0.94 |
| Succinaldehyde-Lys | C_10_H_18_N_2_O_3_ | 8.07 | 214.1317 | 214.1316 | 0.47 |
| 2-Pentenedial-Lys | C_11_H_18_N_2_O_3_ | 12.38 | 226.1317 | 226.1325 | -3.54 |
| Glutaraldehyde-Gln | C_10_H_16_N_2_O_4_ | 9.14 | 228.1110 | 228.1111 | -0.44 |
| Benzaldehyde-Gln | C_12_H_14_N_2_O_3_ | 8.93 | 234.1004 | 234.1003 | 0.43 |
| 4-Hydroxybenzaldehyde-Asn | C_11_H_12_N_2_O_4_ | 4.24 | 236.0797 | 236.0796 | 0.42 |
| 2-Octenal-Asn | C_12_H_20_N_2_O_3_ | 10.35 | 240.1474 | 240.1468 | 2.50 |
| 4-HHE-Gln | C_11_H_18_N_2_O_4_ | 10.51 | 242.1267 | 242.1268 | -0.41 |
| 5-Oxohexanal-Lys | C_12_H_22_N_2_O_3_ | 6.03 | 242.1630 | 242.1630 | 0.00 |
| 2-Phenylpropanal-Asn | C_13_H_16_N_2_O_3_ | 18.21 | 248.1161 | 248.1159 | 0.81 |
| 2-Furancarboxaldehyde-Arg | C_11_H_16_N_4_O_3_ | 10.15 | 252.1222 | 252.1222 | 0.00 |
| Methylimidazole acetaldehyde-Lys | C_12_H_20_N_4_O_2_ | 10.27 | 252.1586 | 252.1586 | 0.00 |
| 2-Nonenal-Asn | C_13_H_22_N_2_O_3_ | 13.42 | 254.1630 | 254.1633 | -1.18 |
| Octanal-Gln | C_13_H_24_N_2_O_3_ | 11.87 | 256.1787 | 256.1781 | 2.34 |
| 4-Hydroxyphenylacetaldehyde-Lys | C_14_H_20_N_2_O_3_ | 11.53 | 264.1474 | 264.1473 | 0.38 |
| Nonanal-Lys | C_15_H_30_N_2_O_2_ | 29.20 | 270.2307 | 270.2307 | 0.00 |
| Cuminaldehyde-Gln | C_15_H_20_N_2_O_3_ | 18.08 | 276.1474 | 276.1471 | 1.09 |
| DOPAL-Lys | C_14_H_18_N_2_O_4_ | 6.48 | 278.1267 | 278.1268 | -0.36 |
| 4-HNE-Lys | C_15_H_28_N_2_O_3_ | 6.96 | 284.2100 | 284.2096 | 1.41 |
| Homovanillin-Lys | C_15_H_22_N_2_O_4_ | 10.95 | 294.1580 | 294.1583 | -1.02 |
| 5-Hydroxyindoleacetaldehyde-Lys | C_16_H_21_N_3_O_3_ | 12.09 | 303.1583 | 303.1586 | -0.99 |
| 2-Decenal-Arg | C_16_H_30_N_4_O_2_ | 9.90 | 310.2369 | 310.2369 | 0.00 |
| Methyl galacturonate-Lys | C_13_H_24_N_2_O_8_ | 18.52 | 336.1533 | 336.1543 | -2.97 |

**Tab. S4** Changes in the levels of aldehyde‑protein amino acid residue conjugates in the substantia nigra between the 6‑OHDA group and the sham group at 6 h post-injection.

| Conjugates | Fold Change | log_2_(FC) | P-value | Trend |
| --- | --- | --- | --- | --- |
| Acrolein-Lys | 3.7606 | 1.9110 | 5.2834E-06 | Up |
| Crotonaldehyde-Lys | 3.2372 | 1.6948 | 4.5868E-08 | Up |
| Benzaldehyde-Gln | 1.0352 | 0.0499 | 0.3499 | Not |
| 4-Hydroxybenzaldehyde-Asn | 3.3858 | 1.7595 | 1.2807E-06 | Up |
| Glyoxal-Lys | 4.0521 | 2.0187 | 4.2583E-05 | Up |
| MDA-Lys | 3.8781 | 1.9553 | 0.0001 | Up |
| Succinaldehyde-Lys | 2.0621 | 1.0441 | 3.5556E-09 | Up |
| Propanal-Asn | 6.4642 | 2.6925 | 5.9473E-08 | Up |
| Octanal-Gln | 2.8196 | 1.4955 | 1.9996E-07 | Up |
| 2-Phenylpropanal-Asn | 1.0670 | 0.0936 | 0.3054 | Not |
| Nonanal-Lys | 2.5766 | 1.3655 | 4.0878E-06 | Up |
| DOPAL-Lys | 1.9784 | 0.9843 | 4.6816E-09 | Up |
| Formaldehyde-Lys | 4.1049 | 2.0373 | 0.0001 | Up |
| Lactaldehyde-Asn | 3.9950 | 1.9982 | 6.9640E-06 | Up |
| Malonic semialdehyde-Asn | 6.6789 | 2.7396 | 1.6427E-08 | Up |
| Cuminaldehyde-Gln | 1.6667 | 0.7370 | 0.0006 | Up |
| 2-Butenedial-Gln | 3.1038 | 1.6340 | 8.3738E-07 | Up |
| 2-Furancarboxaldehyde-Arg | 2.5611 | 1.3568 | 7.8908E-07 | Up |
| 2-Pentenedial-Lys | 2.2316 | 1.1581 | 9.0544E-05 | Up |
| Glutaraldehyde-Gln | 1.9980 | 0.9985 | 3.5821E-07 | Up |
| Methylimidazole acetaldehyde-Lys | 2.6908 | 1.4280 | 8.3461E-06 | Up |
| 2-Octenal-Asn | 2.2715 | 1.1836 | 6.2696E-07 | Up |
| Methyl galacturonate-Lys | 0.9969 | -0.0045 | 0.9723 | Not |
| 5-Oxohexanal-Gln | 2.4597 | 1.2985 | 4.1184E-05 | Up |
| Butanal-Gln | 1.0548 | 0.0770 | 0.5996 | Not |
| 4-Hydroxyphenylacetaldehyde-Lys | 2.7621 | 1.4658 | 1.0961E-06 | Up |
| 2-Nonenal-Asn | 1.0781 | 0.1084 | 0.1903 | Not |
| 2-Decenal-Arg | 1.0202 | 0.0289 | 0.8624 | Not |
| Homovanillin-Lys | 1.9352 | 0.9525 | 2.0033E-06 | Up |
| Pyruvaldehyde-Asn | 2.8717 | 1.5219 | 6.7273E-06 | Up |
| 4-HNE-Lys | 1.7199 | 0.7823 | 5.4689E-05 | Up |
| 4-HHE-Gln | 2.5789 | 1.3667 | 1.2830E-06 | Up |
| 5-Hydroxyindoleacetaldehyde-Lys | 2.5032 | 1.3238 | 4.6125E-09 | Up |

**Tab. S5** Changes in the levels of aldehyde‑protein amino acid residue conjugates in the substantia nigra between the rotenone group and the sham group at 3 d post-injection.

| Conjugates | Fold Change | log_2_(FC) | P-value | Trend |
| --- | --- | --- | --- | --- |
| Acrolein-Lys | 1.5365 | 0.6196 | 1.3658E-03 | Up |
| Crotonaldehyde-Lys | 1.6256 | 0.7010 | 6.9857E-02 | Up |
| Benzaldehyde-Gln | 1.0365 | 0.0517 | 3.6580E-01 | Not |
| 4-Hydroxybenzaldehyde-Asn | 1.3654 | 0.4493 | 1.6580E-03 | Up |
| Glyoxal-Lys | 1.0368 | 0.0521 | 6.9830E-01 | Not |
| MDA-Lys | 1.3011 | 0.3798 | 1.3265E-01 | Up |
| Succinaldehyde-Lys | 0.9687 | -0.0459 | 6.3250E-01 | Not |
| Propanal-Asn | 1.5682 | 0.6491 | 1.5452E-05 | Up |
| Octanal-Gln | 1.0236 | 0.0337 | 1.6520E-01 | Not |
| 2-Phenylpropanal-Asn | 0.9647 | -0.0518 | 2.3690E-01 | Not |
| Nonanal-Lys | 1.8369 | 0.8773 | 3.2540E-02 | Up |
| DOPAL-Lys | 1.5565 | 0.6383 | 1.2365E-05 | Up |
| Formaldehyde-Lys | 1.0236 | 0.0337 | 5.3620E-01 | Not |
| Lactaldehyde-Asn | 1.3658 | 0.4497 | 1.5687E-03 | Up |
| Malonic semialdehyde-Asn | 1.8562 | 0.8924 | 4.2654E-05 | Up |
| Cuminaldehyde-Gln | 1.2656 | 0.3398 | 2.3658E-02 | Up |
| 2-Butenedial-Gln | 1.1145 | 0.1564 | 2.6980E-01 | Not |
| 2-Furancarboxaldehyde-Arg | 1.6987 | 0.7644 | 2.3654E-04 | Up |
| 2-Pentenedial-Lys | 2.0356 | 1.0255 | 3.5680E-07 | Up |
| Glutaraldehyde-Gln | 1.1325 | 0.1795 | 2.3140E-01 | Not |
| Methylimidazole acetaldehyde-Lys | 1.1245 | 0.1693 | 8.7612E-02 | Not |
| 2-Octenal-Asn | 1.4147 | 0.5005 | 8.6597E-02 | Up |
| Methyl galacturonate-Lys | 0.9878 | -0.0177 | 1.3680E-01 | Not |
| 5-Oxohexanal-Gln | 1.1123 | 0.1535 | 3.6899E-01 | Not |
| Butanal-Gln | 0.9878 | -0.0177 | 1.2650E-01 | Not |
| 4-Hydroxyphenylacetaldehyde-Lys | 1.356 | 0.4394 | 1.5687E-02 | Up |
| 2-Nonenal-Asn | 1.0987 | 0.1358 | 3.9874E-01 | Not |
| 2-Decenal-Arg | 0.9647 | -0.0518 | 1.1120E-01 | Not |
| Homovanillin-Lys | 1.0596 | 0.0835 | 2.3540E-01 | Not |
| Pyruvaldehyde-Asn | 1.3239 | 0.4048 | 6.8970E-02 | Up |
| 4-HNE-Lys | 1.2365 | 0.3063 | 1.2657E-02 | Up |
| 4-HHE-Gln | 0.9458 | -0.0804 | 1.2450E-01 | Not |
| 5-Hydroxyindoleacetaldehyde-Lys | 1.1698 | 0.2263 | 3.5682E-02 | Up |

**Tab. S6** Changes in the levels of aldehyde‑protein amino acid residue conjugates in the substantia nigra between the rotenone group and the sham group at 7 d post-injection.

| Conjugates | Fold Change | log_2_(FC) | P-value | Trend |
| --- | --- | --- | --- | --- |
| Acrolein-Lys | 2.1254 | 1.0877 | 5.2356E-05 | Up |
| Crotonaldehyde-Lys | 3.0263 | 1.5976 | 1.5868E-08 | Up |
| Benzaldehyde-Gln | 0.9568 | -0.0637 | 5.2145E-01 | Not |
| 4-Hydroxybenzaldehyde-Asn | 2.2146 | 1.1470 | 5.2425E-05 | Up |
| Glyoxal-Lys | 1.2319 | 0.3009 | 6.0125E-03 | Up |
| MDA-Lys | 2.5470 | 1.3488 | 5.2422E-05 | Up |
| Succinaldehyde-Lys | 3.1248 | 1.6438 | 1.2357E-08 | Up |
| Propanal-Asn | 4.0235 | 2.0085 | 2.3650E-09 | Up |
| Octanal-Gln | 1.6592 | 0.7305 | 3.0255E-05 | Up |
| 2-Phenylpropanal-Asn | 1.0175 | 0.0250 | 5.6320E-01 | Not |
| Nonanal-Lys | 3.1471 | 1.6540 | 1.2536E-05 | Up |
| DOPAL-Lys | 3.5486 | 1.8272 | 4.5785E-08 | Up |
| Formaldehyde-Lys | 1.2655 | 0.3397 | 2.5000E-03 | Up |
| Lactaldehyde-Asn | 2.1494 | 1.1039 | 3.5240E-04 | Up |
| Malonic semialdehyde-Asn | 4.5891 | 2.1982 | 3.5500E-09 | Up |
| Cuminaldehyde-Gln | 1.6984 | 0.7642 | 3.6580E-06 | Up |
| 2-Butenedial-Gln | 1.3696 | 0.4538 | 4.2369E-05 | Up |
| 2-Furancarboxaldehyde-Arg | 2.6871 | 1.4261 | 1.2436E-06 | Up |
| 2-Pentenedial-Lys | 3.1562 | 1.6582 | 1.2370E-05 | Up |
| Glutaraldehyde-Gln | 1.1263 | 0.1716 | 9.2605E-02 | Not |
| Methylimidazole acetaldehyde-Lys | 1.6369 | 0.7110 | 2.5421E-04 | Up |
| 2-Octenal-Asn | 3.1242 | 1.6435 | 2.4520E-07 | Up |
| Methyl galacturonate-Lys | 1.1595 | 0.2135 | 2.3520E-01 | Not |
| 5-Oxohexanal-Gln | 0.9687 | -0.0459 | 1.2347E-01 | Not |
| Butanal-Gln | 1.0265 | 0.0377 | 2.3690E-01 | Not |
| 4-Hydroxyphenylacetaldehyde-Lys | 2.2142 | 1.1468 | 1.0255E-06 | Up |
| 2-Nonenal-Asn | 1.1543 | 0.2070 | 3.6980E-01 | Not |
| 2-Decenal-Arg | 1.5695 | 0.6503 | 6.2400E-04 | Up |
| Homovanillin-Lys | 1.1693 | 0.2256 | 1.7546E-01 | Not |
| Pyruvaldehyde-Asn | 2.1576 | 1.1094 | 1.2536E-04 | Up |
| 4-HNE-Lys | 2.1116 | 1.0783 | 2.4521E-05 | Up |
| 4-HHE-Gln | 1.2651 | 0.3393 | 3.3658E-05 | Up |
| 5-Hydroxyindoleacetaldehyde-Lys | 1.9874 | 0.9909 | 2.4687E-06 | Up |

**Tab. S7** Changes in the levels of aldehyde‑protein amino acid residue conjugates in the substantia nigra between the 6‑OHDA+Alda‑1 group and the 6‑OHDA group at 6 h post-injection.

| Conjugates | Fold Change | log_2_(FC) | P-value | Trend |
| --- | --- | --- | --- | --- |
| Acrolein-Lys | 0.4588 | -1.1241 | 0.0001 | Down |
| Crotonaldehyde-Lys | 0.4969 | -1.0089 | 1.6648E-06 | Down |
| Benzaldehyde-Gln | 0.9322 | -0.1013 | 0.3116 | Not |
| 4-Hydroxybenzaldehyde -Asn | 0.4987 | -1.0038 | 3.2622E-05 | Down |
| Glyoxal-Lys | 0.4780 | -1.0650 | 0.0012 | Down |
| MDA-Lys | 0.4756 | -1.0723 | 0.0018 | Down |
| Succinaldehyde-Lys | 0.5996 | -0.7378 | 5.3713E-14 | Down |
| Propanal-Asn | 0.5049 | -0.9860 | 1.8141E-05 | Down |
| Octanal-Gln | 0.4822 | -1.0522 | 3.1944E-06 | Down |
| 2-Phenylpropanal-Asn | 0.8915 | -0.1657 | 0.3918 | Not |
| Nonanal-Lys | 0.4817 | -1.0538 | 3.0697E-05 | Down |
| DOPAL-Lys | 0.6458 | -0.6308 | 8.6364E-09 | Down |
| Formaldehyde-Lys | 1.0445 | 0.0629 | 0.8038 | Not |
| Lactaldehyde-Asn | 0.6459 | -0.6306 | 0.0056 | Down |
| Malonic semialdehyde-Asn | 0.5026 | -0.9926 | 4.8808E-06 | Down |
| Cuminaldehyde-Gln | 0.9413 | -0.0873 | 0.5544 | Not |
| 2-Butenedial-Gln | 0.5967 | -0.7449 | 0.0002 | Down |
| 2-Furancarboxaldehyde-Arg | 0.9238 | -0.1144 | 0.2418 | Not |
| 2-Pentenedial-Lys | 1.0484 | 0.0681 | 0.5265 | Not |
| Glutaraldehyde-Gln | 0.9222 | -0.1169 | 0.1541 | Not |
| Methylimidazole acetaldehyde-Lys | 0.7252 | -0.4636 | 0.0061 | Down |
| 2-Octenal-Asn | 0.7960 | -0.3291 | 0.0078 | Down |
| Methyl galacturonate-Lys | 0.9085 | -0.1384 | 0.1184 | Not |
| 5-Oxohexanal-Gln | 1.0377 | 0.0534 | 0.7147 | Not |
| Butanal-Gln | 0.8438 | -0.2451 | 0.0403 | Not |
| 4-Hydroxyphenylacetaldehyde-Lys | 0.6463 | -0.6297 | 0.0002 | Down |
| 2-Nonenal-Asn | 0.8946 | -0.1607 | 0.0745 | Not |
| 2-Decenal-Arg | 0.8881 | -0.1713 | 0.3318 | Not |
| Homovanillin-Lys | 0.5695 | -0.8124 | 5.4902E-06 | Down |
| Pyruvaldehyde-Asn | 0.6853 | -0.5452 | 0.0039 | Down |
| 4-HNE-Lys | 0.4948 | -1.0151 | 1.0548E-05 | Down |
| 4-HHE-Gln | 0.4938 | -1.0180 | 6.6467E-06 | Down |
| 5-Hydroxyindoleacetaldehyde-Lys | 0.6513 | -0.6187 | 2.2486E-06 | Down |

**Tab. S8** Changes in the levels of aldehyde‑protein amino acid residue conjugates in the substantia nigra between the 6‑OHDA+phenelzine group and the 6‑OHDA group at 6 h post-injection.

| Conjugates | Fold Change | log_2_(FC) | P-value | Trend |
| --- | --- | --- | --- | --- |
| Acrolein-Lys | 0.6588 | -0.6021 | 0.0052 | Down |
| Crotonaldehyde-Lys | 0.6969 | -0.5209 | 0.0002 | Down |
| Benzaldehyde-Gln | 0.9012 | -0.1501 | 0.1542 | Not |
| 4-Hydroxybenzaldehyde-Asn | 0.7066 | -0.5011 | 0.0032 | Down |
| Glyoxal-Lys | 0.6560 | -0.6083 | 0.0189 | Down |
| MDA-Lys | 0.6634 | -0.5921 | 0.0270 | Down |
| Succinaldehyde-Lys | 0.6930 | -0.5291 | 4.1839E-12 | Down |
| Propanal-Asn | 0.9451 | -0.0814 | 0.5045 | Not |
| Octanal-Gln | 0.9429 | -0.0848 | 0.4166 | Not |
| 2-Phenylpropanal-Asn | 0.9305 | -0.1039 | 0.5130 | Not |
| Nonanal-Lys | 0.9690 | -0.0455 | 0.7227 | Not |
| DOPAL-Lys | 0.7751 | -0.3675 | 2.5310E-06 | Down |
| Formaldehyde-Lys | 0.5893 | -0.7628 | 0.0167 | Down |
| Lactaldehyde-Asn | 0.6561 | -0.6080 | 0.0083 | Down |
| Malonic semialdehyde-Asn | 0.5757 | -0.7967 | 0.0001 | Down |
| Cuminaldehyde-Gln | 0.9703 | -0.0436 | 0.7668 | Not |
| 2-Butenedial-Gln | 0.9968 | -0.0046 | 0.9652 | Not |
| 2-Furancarboxaldehyde-Arg | 1.0612 | 0.0857 | 0.3232 | Not |
| 2-Pentenedial-Lys | 0.9045 | -0.1448 | 0.2157 | Not |
| Glutaraldehyde-Gln | 0.8740 | -0.1942 | 0.1129 | Not |
| Methylimidazole acetaldehyde-Lys | 0.7901 | -0.3400 | 0.0245 | Down |
| 2-Octenal-Asn | 0.9460 | -0.0801 | 0.4339 | Not |
| Methyl galacturonate-Lys | 0.8878 | -0.1716 | 0.2204 | Not |
| 5-Oxohexanal-Gln | 0.6253 | -0.6774 | 0.0015 | Down |
| Butanal-Gln | 0.9045 | -0.1448 | 0.2726 | Not |
| 4-Hydroxyphenylacetaldehyde-Lys | 0.7287 | -0.4567 | 0.0028 | Down |
| 2-Nonenal-Asn | 0.8348 | -0.2604 | 0.0074 | Not |
| 2-Decenal-Arg | 0.8682 | -0.2040 | 0.2548 | Not |
| Homovanillin-Lys | 0.7576 | -0.4005 | 0.0008 | Down |
| Pyruvaldehyde-Asn | 0.7353 | -0.4436 | 0.0117 | Down |
| 4-HNE-Lys | 0.7948 | -0.3314 | 0.0159 | Down |
| 4-HHE-Gln | 0.7438 | -0.4270 | 0.0030 | Down |
| 5-Hydroxyindoleacetaldehyde-Lys | 0.7475 | -0.4199 | 6.8509E-05 | Down |

**Tab. S9** Aldehyde‑modified α‑synuclein peptide fragments in the brain of 6‑OHDA‑treated rats.

| Conjugates | Calculated mass (*m/z*) | Tested mass (*m/z*) | Error (ppm) | Main MS/MS fragments |
| --- | --- | --- | --- | --- |
| Pyruvaldehyde-GLSK | 458.2609 | 458.2595 | 3.06 | 440.2492, 369.2129, 359.1921, 341.1813, 288.1545, 272.1605, 201.1230, 143.1185 |
| 4-Hydroxybenzaldehyde-GLSK | 508.2766 | 508.2760 | 1.18 | 490.2646, 462.2718, 395.1921, 327.2022, 296.1237, 213.1596, 182.0811, 136.0757 |
| Malonic semialdehyde-GLSK | 474.2558 | 474.2576 | -3.80 | 456.2469, 438.2403, 274.1393, 256.1290, 187.1076 |
| Methylimidazole acetaldehyde-GLSK | 510.3035 | 510.3036 | -0.20 | 411.2344, 325.1844, 257.1617, 240.1342, 223.1078, 143.1183 |
| 5-Hydroxyindoleacetaldehyde-GLSK | 561.3031 | 561.3027 | 0.71 | 472.2556, 385.2223, 286.1541, 276.1553, 258.1603, 186.0879 |
| MDA-GLSK | 458.2609 | 458.2598 | 2.40 | 440.2517, 422.2397, 341.1815, 323.1709, 254.1492, 187.1078, 171.1126, 72.0811 |
| 4-Hydroxyphenylacetaldehyde-GLSK | 522.2922 | 522.2916 | 1.15 | 395.1915, 341.2175, 284.1962, 227.1750, 199.1804, 100.1123 |
| DOPAL-GLSK | 536.2715 | 536.2706 | 1.68 | 279.1337, 258.1444, 230.1496, 212.1394, 86.0970, 70.0658 |
| MDA-AK | 272.1605 | 272.1611 | -2.20 | 255.1441, 175.1188, 171.1127, 143.1178, 130.0500, 116.0707, 72.0813 |
| Pyruvaldehyde-AK | 272.1605 | 272.1613 | -2.94 | 255.1441, 175.1189, 158.0925, 143.1179, 112.0871, 72.0814 |
| Octanal-DQMGK | 688.3698 | 688.3669 | 4.21 | 670.3575, 571.2928, 557.2706, 488.2505, 389.1816, 261.1243 |
| Propanal-QGVAEAAGK | 870.4680 | 870.4664 | 1.84 | 853.4366, 739.3746, 670.3557, 571.2857, 488.2499, 389.1819, 280.1639 |
| Crotonaldehyde-AK-TK | 517.3344 | 517.3344 | 0.00 | 430.3005, 402.3046, 317.2180, 204.1342, 173.1282, 147.1126, 129.1022 |
| 4-HNE-AK-AK | 573.3970 | 573.3973 | -0.52 | 399.2968, 373.2807, 314.2075, 260.1971, 201.1235, 173.1285, 147.1130, 129.1024 |
